# Downregulation of the RNA-binding protein PUM2 facilitates MSC-driven bone regeneration and prevents OVX-induced bone loss

**DOI:** 10.1186/s12929-023-00920-8

**Published:** 2023-04-23

**Authors:** Dong Suk Yoon, Yoorim Choi, Kyoung-Mi Lee, Eun Ae Ko, Eun-Ji Kim, Kwang Hwan Park, Jin Woo Lee

**Affiliations:** 1 Department of Biomedical Science, Hwasung Medi-Science University, Hwaseong-Si 18274, Gyeonggi-Do, South Korea; 2 Department of Orthopaedic Surgery, Yonsei University College of Medicine, Seoul, 03722 South Korea; 3 Brain Korea 21 PLUS Project for Medical Science, Yonsei University College of Medicine, Seoul, 03722 South Korea

**Keywords:** Mesenchymal stem cells, PUMILIO2, Osteoporosis, Bone regeneration, Gene therapy

## Abstract

**Background:**

Although mRNA dysregulation can induce changes in mesenchymal stem cell (MSC) homeostasis, the mechanisms by which post-transcriptional regulation influences MSC differentiation potential remain understudied. PUMILIO2 (PUM2) represses translation by binding target mRNAs in a sequence-specific manner.

**Methods:**

In vitro osteogenic differentiation assays were conducted using human bone marrow-derived MSCs. Alkaline phosphatase and alizarin red S staining were used to evaluate the osteogenic potential of MSCs. A rat xenograft model featuring a calvarial defect to examine effects of MSC-driven bone regeneration. RNA-immunoprecipitation (RNA-IP) assay was used to determine the interaction between PUM2 protein and *Distal-Less Homeobox 5* (*DLX5*) mRNA. Ovariectomized (OVX) mice were employed to evaluate the effect of gene therapy for postmenopausal osteoporosis.

**Results:**

Here, we elucidated the molecular mechanism of PUM2 in MSC osteogenesis and evaluated the applicability of PUM2 knockdown (KD) as a potential cell-based or gene therapy. PUM2 level was downregulated during MSC osteogenic differentiation, and PUM2 KD enhanced MSC osteogenic potential. Following PUM2 KD, MSCs were transplanted onto calvarial defects in 12-week-old rats; after 8 weeks, transplanted MSCs promoted bone regeneration. PUM2 KD upregulated the expression of DLX5 mRNA and protein and the reporter activity of its 3'-untranslated region. RNA-IP revealed direct binding of PUM2 to *DLX5* mRNA. We then evaluated the potential of adeno-associated virus serotype 9 (AAV9)-si*Pum2* as a gene therapy for osteoporosis in OVX mice.

**Conclusion:**

Our findings suggest a novel role for PUM2 in MSC osteogenesis and highlight the potential of PUM2 KD-MSCs in bone regeneration. Additionally, we showed that AAV9-si*Pum2* is a potential gene therapy for osteoporosis.

**Supplementary Information:**

The online version contains supplementary material available at 10.1186/s12929-023-00920-8.

## Background

Osteoblast-mediated bone tissue production is one of the most important functions in maintaining normal bone homeostasis [1–3]. The function of osteoblasts declines with age, which complicates bone maintenance and impairs the performance of normal activities in older people [4, 5]. Although osteoporosis arises owing to decreased osteoblast activity and increased osteoclast activity, resulting in loss of homeostasis, most therapeutic drug development studies have been conducted with a little focus on the inhibition of osteoclast-mediated bone resorption [6]. In the aging process, a decrease in the mesenchymal stem cell (MSC) pool and biased differentiation towards an adipogenic lineage reduce the supply of osteoblast precursors, leading to decreased bone formation and the development of osteoporosis [7, 8]. In bone homeostasis, several signaling pathways associated with bone-reducing diseases have been studied for decades. In particular, the undesirable determination of MSC cell fate to the adipogenic lineage is regulated by lineage-specific transcription factors such as runt-related transcription factor 2 (RUNX2) and peroxisome proliferator-activated receptor gamma (PPARγ), which causes an imbalance between osteoblast and adipocytes [8, 9]. It has been shown that these two key transcription factors that play important roles in MSC differentiation can be regulated by Distal-Less Homeobox 5 (DLX5), which is known as an enhancer of *RUNX2* expression and an inhibitor of *PPARγ* expression [10–12]. However, few studies have been conducted on how DLX5 expression is regulated during osteoblast differentiation of MSCs. Therefore, it is essential to investigate the osteogenic differentiation of MSCs to discover novel and functional molecular targets that can improve MSC osteogenic ability for the development of therapeutic candidates related to bone-reducing diseases.

RNA-binding proteins (RBPs) help regulate multiple post-transcriptional characteristics, including the structure, stability, function, and cellular localization of their target mRNAs [13]. Although some RBPs are involved in the MSC osteogenic differentiation process [14, 15], studies on many RBPs and their target RNAs are limited, and more studies are required before their use as targets for gene or cell therapies. PUMILIO1 (PUM1) and PUMILIO2 (PUM2) are RBPs that bind to target mRNAs in a sequence-specific manner using their homology domains, which are composed of eight imperfect tandem repeats [16]. PUM1 and PUM2 recognize conserved 8-nucleotide (UGUA_AUA) response elements in the 3′-untranslated regions (UTRs) of target mRNAs [17]. PUMILIO proteins are involved in biological processes including stem cell maintenance, organismal development, human disease pathogenesis, and ribosome biogenesis [18]. While little is known about the functions of PUMILIO proteins in humans, they appear to have evolutionarily conserved structures and functions [19, 20]. Overexpression of *PUM2* inhibits the osteogenic differentiation of MSCs, and silencing of *Pum2* in zebrafish embryos results in abnormal bone development [15]. *PUM1* silencing affects the osteogenic and chondrogenic differentiation potential of human MSCs [21]. However, mechanistic insights into how PUM1 and PUM2 affect the osteogenic differentiation capacity of MSCs and how to apply PUM1 and PUM2 as targets for the treatment of bone-related diseases are still lacking. Therefore, studies on the utilization of *PUM1* and *PUM2* as therapeutic target genes, and a more in-depth analysis of the molecular mechanisms of PUM1 and PUM2 in regulating the differentiation capacities of MSCs, are needed.

Here, we aimed to identify the role of PUM2 in MSC osteogenesis by investigating its interactions with potential RNA targets. We revealed that knockdown (KD) of PUM2 enhances the osteogenic potential of human bone marrow-derived MSCs. Transplantation of human MSCs with PUM2 KD increased the regenerative capacity of damaged calvarial bone in rats. Mechanistically, we confirmed that PUM2 binds to DLX5 mRNA, downregulates DLX5 expression, and decreases the reporter activity of the DLX5 3’-UTR. Furthermore, the systemic delivery of adeno-associated virus serotype 9 (rAAV9)-si*Pum2* prevented bone loss in an ovariectomized (OVX) animal model of postmenopausal osteoporosis. Our findings can help us assess RBP-mediated control of MSC osteogenesis and allow us to identify potential therapeutic targets for bone-related diseases and prevent osteoporotic bone loss.

## Materials and methods

### *Cellcultureandosteogenicdifferentiation*

Bone marrow aspirates were obtained from the posterior iliac crests of eight adult donors with the approval of the Institutional Review Board (IRB; 2017-0308-001) of the Yonsei University College of Medicine, and informed consent was obtained from all participants. Bone marrow-derived MSCs were selected based on their adherence to plastic cell culture dishes. The protocol for MSC culture has been described previously [22]. MSCs were maintained in low-glucose Dulbecco’s modified Eagle’s medium (DMEM-LG; Invitrogen, Carlsbad, CA, USA) supplemented with 10% fetal bovine serum (FBS; Gibco, Grand Island, NY, USA) and 1% antibiotic–antimycotic solution (Invitrogen) at 37 °C and 5% CO_2_ and were subcultured at 80% confluency. To induce MSC differentiation into the osteogenic lineage, the cells were seeded at 8 × 10^4^ cells/well in 12-well plates and cultured in osteogenic medium [DMEM-LG containing 10% FBS, 1% antibiotic–antimycotic solution, 10 mM β-glycerophosphate (Sigma-Aldrich, St. Louis, MO, USA), and 50 μg/mL ascorbic acid (Gibco)] for 10 days. The medium was replaced every 2 days. Alizarin red S staining was performed to determine the osteogenic capacity of MSCs. After fixation in ice-cold 70% ethanol, 1 mL of freshly prepared 3% alizarin red S solution (wt/vol) (Sigma-Aldrich) was added before incubation in the dark for 30 min. For quantification, the absorbance was detected at 595 nm by destaining with 10% cetylpyridinium chloride monohydrate (Sigma-Aldrich) for 30 min.

### *Westernblotanalysis*

MSCs were lysed in PROPREP™ Protein Extraction Solution (iNtRON Biotechnology, Seongnam, South Korea). The protein concentrations were determined using the Bio-Rad Protein Assay (Bio-Rad Laboratories, Inc., Hercules, CA, USA). Approximately, 30 μg of the protein was analyzed using 10% sodium dodecyl sulfate–polyacrylamide gel electrophoresis (Sigma-Aldrich). The resolved proteins were transferred to membranes and blocked with 5% skim milk (BD, Sparks, MD, USA) for 1 h at room temperature. The membranes were incubated overnight with antibodies against PUM1 (1:1000; Abcam, Cambridge, UK), PUM2 (1:1000; Santa Cruz Biotechnology, Santa Cruz, CA, USA), RUNX2 (1:1000; Santa Cruz Biotechnology), COL1A1 (1:1000; Cell Signaling Technology, Danvers, MA, USA), OPN (1:1000; Santa Cruz Biotechnology), TurboGFP (copGFP, 1:1000; Invitrogen), GFP (1:1000; Santa Cruz Biotechnology), and DLX5 (1:1000; Santa Cruz Biotechnology). A β-actin probe (1:5000; Santa Cruz Biotechnology) served as the loading control. The band intensity on the western blot was quantified using ImageJ (n = 3, in triplicate).

### *Quantitativereversetranscription-polymerasechainreaction(qRT-PCR)*

PCR analysis was performed as described previously [23]. Total RNA was isolated using the RNeasy kit (Qiagen, Valencia, CA, USA), according to the manufacturer’s instructions. Total RNA (1 μg) was reverse-transcribed using the Omniscript kit (Qiagen, Hilden, Germany). The primer sets used in this study (Additional file 1: Table S2) were validated and purchased from Bioneer (Daejeon, South Korea). The mean cycle threshold values from triplicate (n = 3) measurements were used to calculate gene expression, with normalization to ACTB (β-actin) expression. Data were analyzed according to the comparative cycle threshold (Ct) method [24].

### *RNAinterference*

All siRNAs used in this study were purchased from Bioneer (Additional file 1: Table S3). MSCs were plated to obtain 70–80% confluence in 6-well plates and transfected with 100 nM of each siRNA targeting *PUM1* or *PUM2*, or non-targeting control, using Lipofectamine 2000 (Invitrogen). Lipofectamine 2000 was used in 2ul each in 1 ml of cell culture medium. After 6 h of transfection, the medium was replaced. On the next day, the cells were detached from the plates, counted, and replated at appropriate cell densities for further experiments.

### *Humanosteocalcin(OCN)pGreenZeodifferentiationreporterassay*

To establish human MSCs stably expressing copGFP under the activity of *OCN* promoter/enhancer, the lentiviral plasmid vector for the human OCN pGreenZeo differentiation reporter was purchased (System Biosciences, LLC; SBI SR1003PA-1). OCN pGreenZeo differentiation reporter assay was performed as described previously [25]. HEK293T cells were seeded in T75 flasks at 5 × 10^6^ cells per dish to derive lentiviral particles with a human OCN pGreenZeo differentiation reporter. The attached cells were transfected with lentiviral plasmids expressing the human OCN pGreenZeo differentiation reporter, psPAX2 (http://www.addgene.org/12260/) and pMD2.G (http://www.addgene.org/12259/), using the CalFectin mammalian cell transfection reagent (SignaGen Laboratories, Frederick, MD, USA). After about 6 h of transfection, the medium was replaced.The transfected cells were maintained for 2 days, and the supernatants were collected and stored at − 70 °C. To monitor GFP activity in MSCs transduced with lentivirus carrying the OCN pGreenZeo differentiation reporter, the cells were seeded in 6-well plates at 1 × 10^5^ cells under growth or osteogenic conditions. After 10 days, the copGFP signal was observed using fluorescence microscopy. For quantitative analysis, the passive lysis buffer (Promega, Madison, WI, USA) was added to the cells that were incubated at 4 °C for 5 min. The whole cell lysate was transferred to a microcentrifuge tube and centrifuged to collect the supernatant. After determining the total protein concentration of each sample, the fluorescence intensity was measured at 360 nm/465 nm (excitation/emission) using a plate reader.

### *Alkalinephosphatase(ALP)staining*

The method used for ALP staining has been described previously [26]. MSCs transfected with NC siRNA or *PUM2* siRNA were fixed in a 2:3 citrate buffer/acetone fixative. The MSCs were stained for ALP using an alkaline staining solution mixed with fast violet B salt (Sigma-Aldrich) in a naphthol AS-MX phosphate alkaline solution (Sigma-Aldrich) for 30 min in the dark. The stained cells were then rinsed with tap water.

### *ALPactivityassay*

The method used to measure ALP activity has been described previously [26]. NC or *PUM2* siRNA-transfected MSCs were incubated for 30 min at 37 °C in lysis buffer (0.5% Triton X-100, 0.4 mM Tris–HCl [pH 7.5], and 4.5 mM NaCl) and then collected by pipetting. After centrifugation at 13,000 rpm at 4 °C for 10 min, ALP enzyme activity was measured using the supernatant obtained from triplicate cultures. An ALP substrate kit (Sigma-Aldrich) was used according to the manufacturer’s instructions. The absorbance was measured at 405 nm using a spectrophotometer. ALP activity was normalized to the total protein content in each sample and expressed as μmol/mL of total protein.

### *Calvarialdefects*

All animal procedures were conducted in accordance with the Institutional Animal Care and Use Committee (IACUC) protocol of Yonsei University College of Medicine (IACUC-2016-0099). Twelve-week-old male Sprague–Dawley rats were anesthetized by intraperitoneal injection of zoletile (30 mg/kg body weight) and rumpon (10 mg/kg body weight). After shaving the fur on the head, a longitudinal incision was made in the skull, and then an 8 mm-diameter critical-size calvarial bone defect was created using a trephine bur. The defects were irrigated with saline, and MSCs (1 × 10^6^ per defect) mixed with fibrin glue were implanted into the defects, followed by soft tissue suturing. For pain relief, the rats received a subcutaneous injection of metacam (meloxicam, 0.2 mg/kg). At postoperative week 8, the rats were sacrificed, and their skulls were harvested for µCT and histological analysis (n = 9 per group).

### *Micro-computedtomography(µCT)*

After fixation in 10% formalin for 1 week, the skulls were scanned using a Skyscan 1076 high-resolution µCT machine (Bruker, Billerica, MA, USA) to quantify calvarial bone regeneration at the site of the defect. The images were reconstructed and analyzed using NRecon v1.6.6.0 (Bruker) and CTAn v1.13.2.1. CTVol v2.0 three-dimensional visualization software (Bruker) was used to analyze the regeneration of the calvarial bone. The X-ray source settings were a voltage of 70 kVp and a current of 140 mA. A 0.5 mm-thick aluminum filter was used for beam induration. The pixel size was 18 mm, the exposure time was 1475 ms, and the rotation step was 0.5°, with a complete rotation of over 360°.

### *Histologyandimmunohistochemistry*

Repaired bone tissue sections were fixed for 7 days in 10% formalin at room temperature. After fixation, formalin-fixed specimens were embedded in paraffin. The sections were deparaffinized, rehydrated, and washed with PBS and then used to evaluate bone regeneration in the damaged regions. The prepared bone tissue samples were sliced 4 mm-thick and stained with hematoxylin and eosin to observe bone healing or incubated with human anti-vimentin (Santa Cruz Biotechnology), OPN (Abcam), OCN (Abcam), and CD4 (Abcam) antibodies to confirm whether the regenerated bone tissue had a human origin. The stained tissues were observed using a VS120 virtual microscope (Olympus, Tokyo, Japan), and the images were analyzed using OlyVIA 2.5 (Olympus).

### RNA-immunoprecipitation

The methodology for RNA-immunoprecipitation (RNA-IP) was described previously [21]. RNA-IP was performed to determine the potential interactions between PUM2 and *DLX5* mRNA. MSCs (1 × 10^7^) transfected with the pEGFP-C1-PUM2 vector were used in each experiment. PUM2-bound RNA-IP was performed using the RiboCluster Profiler RIP assay kit protocol (MBL International Corporation, Woburn, MA, USA). The cells were lysed using dithiothreitol (DTT; Sigma)-added lysis buffer from the manufacturer, and then the lysates were precleared with protein G plus agarose beads (Thermo Fisher Scientific) in DTT-added wash buffer from the manufacturer. The pre-cleared lysates were transferred to tubes containing GFP antibody (Santa Cruz Biotechnology) or IgG-immobilized beads. After overnight incubation, GFP antibody or IgG-immobilized protein G agarose beads-RNA/protein complexes were separated to extract proteins and PUM2-bound RNAs. The eluted RNA was analyzed using quantitative polymerase chain reaction (qPCR). GFP antibody-bound RNAs were isolated and purified using a kit according to the manufacturer’s instructions.

### *VectorpreparationandtransfectionofplasmidDNAusingelectroporation*

The PUM2 gene was synthesized using the cloning services of AbClon (Seoul, South Korea) and cloned into pcDNA between the NheI and XhoI sites (Takara Bio, Inc., Shiga, Japan) to generate pcDNA-PUM2 that expresses the PUM2-HA protein or pEGFP-C1 vector between the NheI and AgeI sites (Takara Bio, Inc.) to generate pEGFPC1-PUM2, which expressed a GFP-PUM2 fusion protein. A mock vector was used as the control. The PUM2 mutant lacking the RNA-binding domain (ΔPUM2-HD) was cloned into the pEGFP-C1 vector between the NheI and XhoI sites (Takara Bio, Inc.) to generate pEGFPC1-ΔPUM2-HD, which expressed a GFP-PUM2 fusion protein (see Additional file 1: Fig. S1). Each vector was transfected into MSCs using electroporation with the neon transfection system, according to the manufacturer’s instructions (Cat no., MPK5000; Invitrogen). After transfection of the plasmids, the cells were washed with PBS and detached using trypsin/EDTA solution. Approximately, 1 × 10^6^ cells were suspended in 90 µL of Transfection Resuspension Buffer R and mixed with 5 μg of plasmid DNA in a 10 µL volume. Next, a 100 µL aliquot was placed in the electroporator Neon Gold Pipette Tips provided in the kit and pulsed (voltage: 990 V, pulse width: 40 ms). The contents of the transfection pipette were placed in a 100 mm cell culture dish. Transfection efficiency was confirmed by fluorescence microscopy and western blot analysis 24 h after electroporation.

### *DLX53’-UTRGFPreporterassay*

DLX5 3’-UTR lentiviral reporter-GFP vector (Cat. No. 182990810295, NM_005221.6) was purchased commercially from Applied Biological Materials Inc. (Richmond, BC, Canada). We also made point mutation of the DLX5 3’-UTR lentiviral reporter-GFP vector to the three putative PUM2 binding sites of the vector and confirmed the mutated plasmid by sequencing (see Additional file 1: Fig. S2). To obtain lentiviral particles with DLX5 3’UTR-reporter-GFP, HEK293T cells were seeded in 100 mm culture dishes at 3 × 10^6^ cells per dish. On Tthe next day, the cells were transfected with DLX5 3’-UTR Lenti-reporter-GFP vector with psPAX2 (Addgene #12260) and pMD2.G (Addgene #12259) using Lipofectamine 2000 (Invitrogen). After 6 h of transfection, the culture medium was replaced. The lentiviral vector-transfected HEK293T cells were maintained for 2 days, and subsequently, the supernatants were collected and stored at − 70 °C. To transduce the vector expressing DLX5 3’UTR-reporter-GFP, MSCs were seeded in 6-well plates at 5 × 10^4^ cells/well. After 48 h of transduction, the medium was replaced with 10 μg/mL puromycin dihydrochloride (Sigma), and the cells were maintained for 3 days. The selected MSCs were used in the DLX5 3’-UTR GFP reporter assay. The pcDNA3-mock, pcDNA3-PUM2::HA, pEGFP-C1-mock, pEGFP-C1-PUM2, or pEGFP-C1-ΔPUM2-HD vectors were transfected into the selected cells using puromycin dihydrochloride and Lipofectamine 2000 and maintained in growth medium for 2 days, after which the cells were lysed using PRO-PREP Protein Extraction Solution. The protein was analyzed using western blotting to compare the GFP activities of the DLX5 3’-UTR. Western blot band intensity was quantified using ImageJ (n = 3, in triplicate).

### *Ovariectomized(OVX)miceandAAV9-siPum2injection*

The Committee on the Ethics of Animal Experiments of Yonsei University College of Medicine approved all animal experiments and protocols (IACUC-2020-0045). Female sham-operated and OVX mice (C57BL/6 J, 6 weeks old) were purchased from Japan SLC (Hamamatsu, Shizuoka, Japan). Mouse *PUM2* AAV siRNA-pooled virus (serotype 9) was purchased from Abm (Cat. no. 382060940219; Applied Biological Materials Inc., Richmond, BC, Canada). The AAV injection protocols have been described previously [27]. Sham or OVX mice were intravenously injected (IV injection) with 200 μl of AAV9 carrying NC siRNA (approximately 2.5 × 10^10^ GC/mL) or mouse *PUM2*-targeting siRNA (approximately 2.6 × 10^10^ GC/mL). Twelve weeks after the injection, the mice were killed for further analysis. To confirm whether AAV9 carrying NC siRNA or mouse *PUM2*-targeting siRNA was transduced into the bone tissues, the collected samples were visualized using an animal optical imaging system (IVIS, Caliper Life Sciences, MA, USA). Scanning was performed under anesthesia at wavelengths of 744 and 805 nm. The fluorescence intensity was calculated from the fluorescence signal (p/s/cm^2^) using the Living Image software version 2.50 (Xenogen). To ensure reproducibility, all tests were performed on at least three mice (see Additional file 1: Fig. S3). Femur samples were fixed for µCT analysis and histology.

### Statistics and reproducibility

Analyses were performed using one-way ANOVA or Student’s t-test (GraphPad Prism 6). Data are presented as the mean ± standard deviation for at least three individual experiments. All experiments included at least three biological replicates, and the number of replicates is indicated in the text or figure legends.

### Data availability

The datasets used and/or analyzed here are included in this published article or are available from the corresponding author upon request.

## Results

### *PUM2silencingenhancestheosteogenicpotentialofMSCs*

**Fig. 1:**
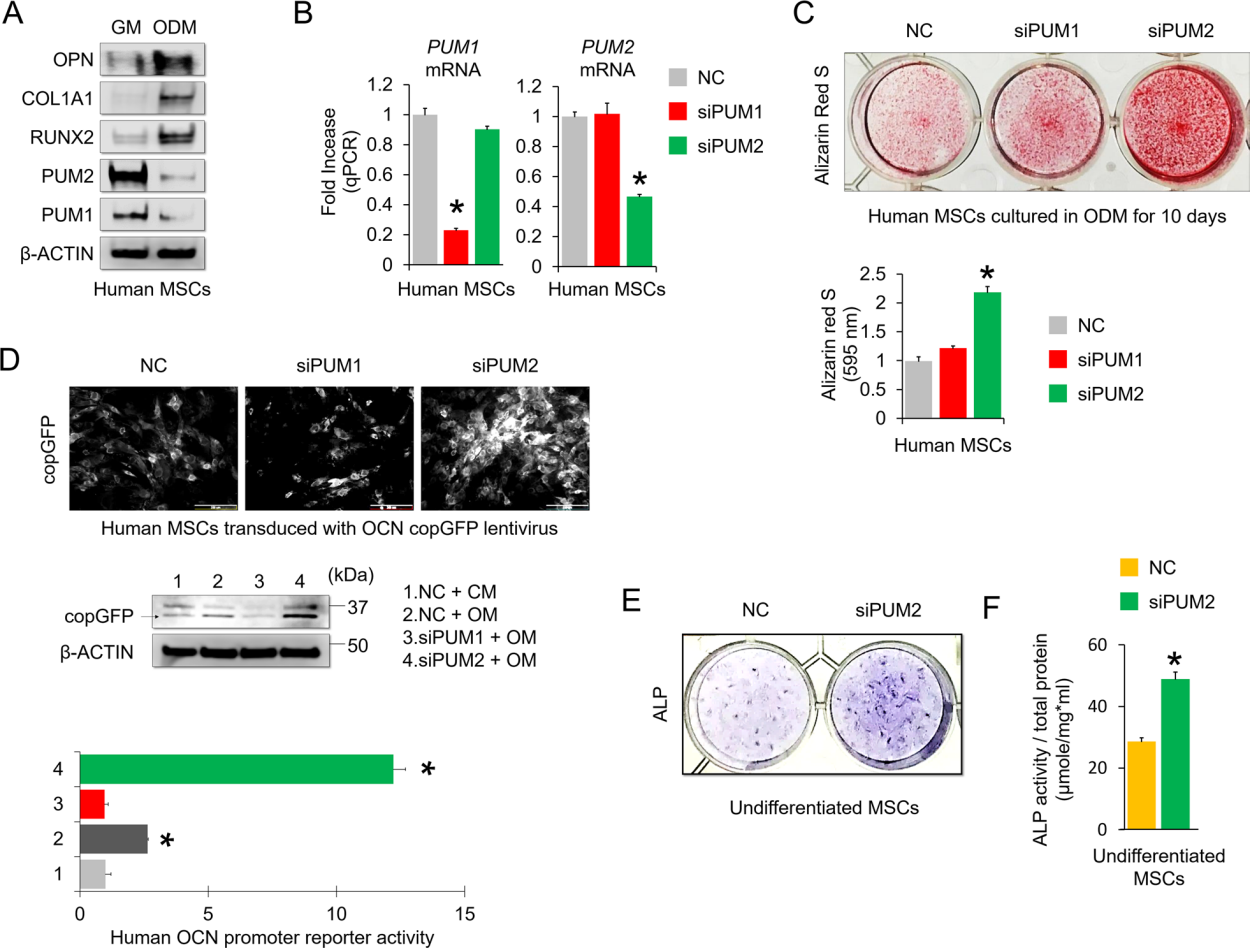
Knockdown of PUM2 promotes the in vitro osteogenic potential of MSCs. **A** Representative images of western blot analysis for protein levels of PUM1, PUM2, RUNX2, COL1A1, and OPN in MSCs that were cultured with growth medium (GM) or osteogenic differentiation medium (ODM) for 10 days. β-ACTIN was used as a loading control. **B** qRT-PCR analysis of *PUM1* and *PUM2* mRNA expression in MSCs that were transfected with non-targeting control (NC), *PUM1*, or *PUM2* siRNA. *P < 0.05 in comparison with NC group (n = 3 experimental replicates). **C** Representative images of alizarin red S staining in NC, *PUM1*, or *PUM2* siRNA-transfected MSCs cultured with ODM for 10 days. For quantification, absorbance was measured at 595 nm following destaining with 10% cetylpyridinium for 30 min. *P < 0.05 compared with NC group (n = 3 experimental replicates). **D** Fluorescence and western blot images showing the activity of *OCN* promoter and the *OCN* promoter reporter quantity. MSCs were transduced with lentivirus of OCN pGreenZeo differentiation reporter, the transduced MSCs were then transfected with NC, *PUM1*, or *PUM2* siRNA. The bar graph indicates the fluorescence-based activities of the OCN promoter. *P < 0.05 compared with the NC group (n = 3 experimental replicates). OCN, osteocalcin; copGFP, copepod green fluorescence protein. Scale bar = 150 μm. **E** ALP staining was performed to evaluate whether the *PUM2* siRNA-transfected MSCs are committed to the osteogenic lineage. Staining was performed using undifferentiated MSCs on day 4 post-transfection. This experiment was performed (n = 3 experimental replicates), and representative data are shown. **F** Likewise, the MSCs, on 4 days of post-transfection, were harvested to measure ALP activity. The results for ALP activity were normalized to the amount of total protein. *P > 0.05 compared with NC (n = 3 experimental replicates)

To investigate whether PUM1 and PUM2 are involved in the osteogenic differentiation of human MSCs, we performed western blot analysis using proteins from human bone marrow-derived MSCs grown in growth or differentiation media for 14 days. PUM1 and PUM2 levels were low in MSCs cultured in osteogenic medium, whereas protein levels of osteogenic markers, including runt related transcription factor 2 (RUNX2), collagen type I alpha 1 chain (COL1A1), and osteopontin (OPN), were higher in MSCs cultured in osteogenic medium than in those cultured in growth medium (Fig. 1A). Next, we used small interfering RNA (siRNA)-mediated KD of PUM1 or PUM2 to determine whether a decrease in PUM1 or PUM2 protein levels affected the osteogenic potential of MSCs (Fig. 1B). Alizarin red S staining showed that PUM2 KD increased the osteogenic potential of MSCs cultured in osteogenic medium for 10 days, whereas PUM1 KD did not affect MSC osteogenic potential (Fig. 1C). We recently reported that PUM1 silencing significantly affects the senescence and proliferative capacity of human mesenchymal stem cells and chondrocytes [21]. On the other hand, PUM2 silencing had little effect on cellular senescence and proliferation of MSCs and chondrocytes. However, silencing PUM1 on mid-passage MSCs using siRNA further accelerates senescence in cells, resulting in loss of most of their differentiation potentials due to functional decline due to cell senescence. In the case of the current study, PUM2 was transiently knocked down in early-passage MSCs to confirm only the effect on the differentiation potential. As a result, it was confirmed that PUM2 silencing could have a more dramatic effect on the osteogenic potential of MSCs than PUM1 silencing, whereas si-RNA-mediated knockdown of PUM1 did not show a significant change in the osteogenesis potential of MSCs. For this reason, we conclude that PUM1 does not directly affect the osteogenic potential of MSCs and only PUM2 was considered in subsequent experiments. We confirmed that PUM2 KD enhanced the promoter activity of human osteocalcin (*OCN*) in MSCs cultured in osteogenic medium, whereas PUM1 KD reduced *OCN* promoter activity (Fig. 1D). ALP staining and activity assays were performed to clarify whether MSCs with PUM2-KD could be committed to an osteogenic lineage, even in an undifferentiated state [28]. The number of ALP-positive cells and their activities were significantly higher in PUM2 siRNA-transfected MSCs than in control cells (Fig. 1E, F). Fluorescence-activated cell sorting (FACS) revealed differences in the levels of ALP between the two groups, with values of 12.99% ± 2.71 in negative control (NC) siRNA-transfected MSCs and 28.09% ± 3.49 in PUM2 siRNA-transfected MSCs (Additional file 1: Fig. S4A and S4B). Therefore, we conclude that PUM2 is a repressor of MSC osteogenesis and that a decrease in PUM2 expression is implicated in the commitment of MSCs to the osteogenic lineage.

### *TransplantationofPUM2KDMSCspromotestheefficiencyofboneregenerationincalvarialbonedefects*

**Fig. 2:**
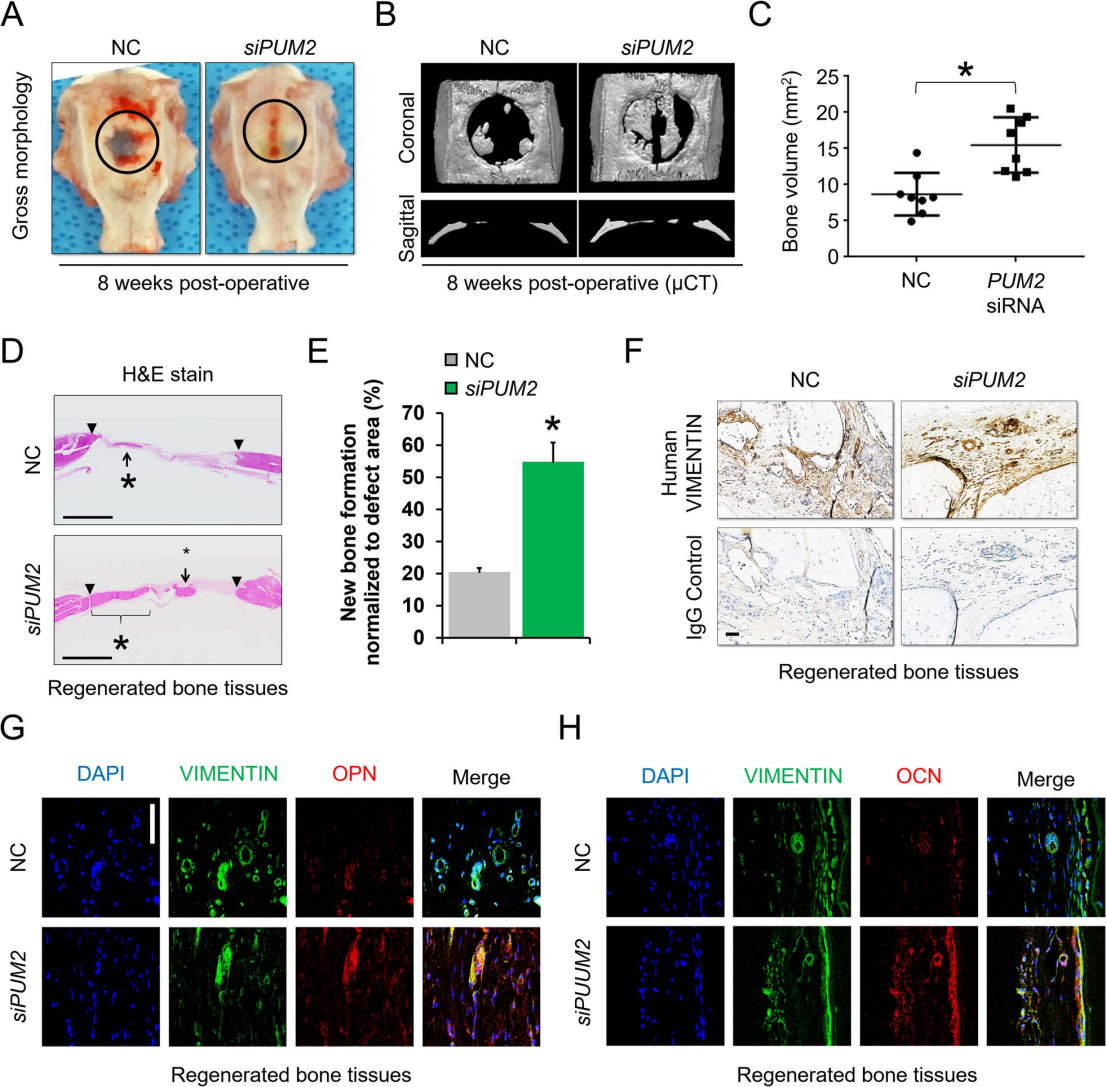
Transplantation of MSCs with *PUM*-KD enhances in vivo bone regeneration in a rat calvarial defect model. **A** The calvarial defect sites of the NC and *PUM2* siRNA groups at 8 weeks post-transplantation of the siRNA-transfected MSCs mixed with fibrin glue (n = 8 for each group). **B** µCT images show bone regeneration at calvarial defect sites 8 weeks after the implantation of NC or *PUM2* siRNA-transfected MSCs mixed with fibrin glue (n = 8 for each group). **C** The graph shows the regenerated bone volume per mm^2^ (n = 8 for each group). *P < 0.05 compared with NC. *P*-values were calculated using ANOVA. Results are expressed as the mean and the error bars denote standard deviation. µCT, microcomputed tomography. **D**, **E** Hematoxylin and eosin staining was performed to observe new bone formation. The arrowheads show the edges of the host bone and line with asterisks and arrows indicates newly regenerated bone. Scale bar = 300 μm. The graph shows new bone formation normalized to defect area (n = 8 for each group). *P < 0.05 compared with NC. **F** To confirm whether the newly regenerated bone tissues were derived from a human origin, immunohistochemistry (IHC) was performed using antibodies specific to human vimentin. Brown tissue represents tissue derived from a human origin (n = 8 for each group). Scale bar = 20 μm. **G**, **H** To confirm whether the transplanted MSCs contributed to new bone formation of calvarial defects, IHC was performed using antibodies against OPN and OCN, as well as antibodies specific to human vimentin. The nuclei were stained with DAPI, and human vimentin was stained with FITC-conjugated secondary antibody. OPN and OCN were stained with phycoerythrin (PE, red)-conjugated secondary antibody (n = 8 for each group). Scale bar = 50 μm

To verify our in vitro finding that knockdown of PUM2 enhances bone healing via MSCs, we employed a rat xenograft model featuring a calvarial defect to examine whether the KD of PUM2 affected MSC-driven bone regeneration. Before transplantation, MSCs transfected with NC or PUM2 siRNA were detached from cell culture plates, mixed with fibrin gel, and then transplanted onto the calvarial defects of 12-week-old rats. After 8 weeks, the transplants were harvested and subjected to X-ray micro-computed tomography (µCT) to observe the gross morphology of the covered tissues in the calvarial defects. In the PUM2 KD group, the surface of the defect site was almost filled with bone-like tissue similar to the surrounding bone. However, this was not observed in the NC group (Fig. 2A). µCT examination revealed enhanced bone regeneration in the group transplanted with PUM2 siRNA-transfected MSCs compared with that in the control group (Fig. 2B). The *PUM2* siRNA group (16.20 ± 0.54, n = 8) showed superior bone regeneration compared to the NC siRNA group (13.47 ± 0.42, n = 8) (Fig. 2C). Hematoxylin and eosin-stained sections showed newly formed bone tissue 8 weeks after transplantation. In the NC RNAi group, a small amount of new bone was observed within the defect region, and most of the defect areas were covered by fibrous tissue (Fig. 2D, upper). In the PUM2 RNAi group, the bone surface of the defect site was almost filled with bone-like tissues, similar to the surrounding normal bone (Fig. 2D, lower). To confirm whether the fibrin glue used for the purpose of MSC delivery in this study could have its own bone regeneration ability, a group injected with fibrin glue and a group without injection were compared. As a result, it was confirmed that the rat calvarial defect did not self-regenerate using the fibrin glue alone (Additional file 1: Fig. S5). Almost no infiltration of immune cells was observed in the area where bone tissue was regenerated in the site where human MSCs were transplanted (Additional file 1: Fig. S6), which is thought to be because MSCs have an immunomodulatory effect [29]. New bone formation was significantly higher in the PUM2 RNAi group than in the NC RNAi group (Fig. 2E). The defects were stained for human-specific vimentin to confirm whether the transplanted human MSCs or host rat cells contributed to new bone formation. Whether the antibody used in this study has specificity only for human cells was confirmed through western blot analysis, and it was confirmed that there was no specificity in rat MSCs (Additional file 1: Fig. S7). Newly formed bone tissues were mostly positive for vimentin (Fig. 2F). To explain the discrepancy concerning the greater bone regenerative capacity in the PUM2 siRNA-treated group than that in the control group, we performed immunostaining for OPN and OCN, which are downstream target genes of RUNX2 [30]. Both samples were cross-stained for human vimentin. The levels of OPN and OCN in the PUM2 siRNA-transfected group were higher than those in the NC siRNA-transfected group and the samples were positive for human vimentin (Fig. 2G, H). Therefore, the transplanted human MSCs successfully differentiated into the osteogenic lineage in the rat calvarial defect xenograft model. Thus, *PUM2* may be a target for enhancing the efficiency of bone healing. We also confirmed that *Pum2* knockdown can enhance the osteogenic differentiation potential of rat MSCs, predicting the functional preservation of PUM2 across species (Additional file 1: Fig. S8).

### *PUM2negativelyregulatesDLX5mRNAexpression*

**Fig. 3:**
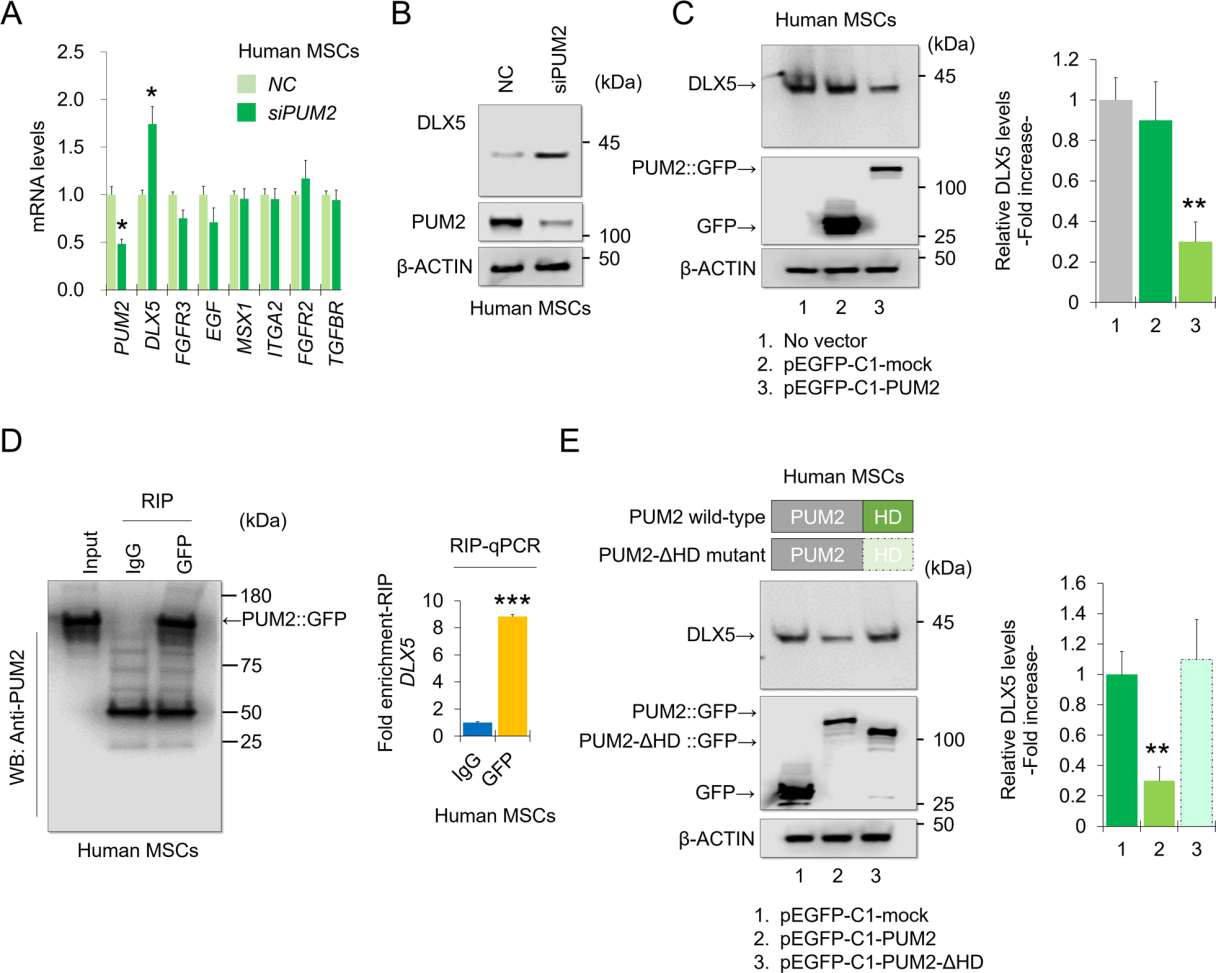
PUM2 negatively regulates mRNA expression of DLX5. **A** qPCR data using RNA from MSCs transfected with siRNA targeting *PUM2*, compared to the group transfected with negative control (NC) siRNAs (n = 3 experimental replicates). **B** PUM2 and DLX5 protein levels were analyzed by western blotting for MSCs transfected with NC or *PUM2* siRNA. **C** PUM2::GFP and DLX5 protein levels were analyzed in MSCs using western blotting for cells transfected with the pEGFP-C1 vector control or the pEGFP-C1-PUM2 vector. **D** PUM2::GFP-immunoprecipitation was successful; the bar graph displays the RIP-qPCR fold enrichment of mRNAs relative to the IgG control. *P < 0.05 in a comparison with the IgG control (n = 3 experimental replicates). **E** GFP and DLX5 levels were analyzed in MSCs using western blotting after transfection with the pEGFP-C1 vector control, pEGFP-C1-PUM1, or pEGFP-C1-PUM1 mutant vector (PUM2-ΔHD)

Bohn et al. identified functional target RNAs of human PUM1 and PUM2 in HEK293 cells using RNA-sequencing [31] (See also Additional file 1: method). From these results, we deduced candidate target genes of PUM2 during MSC osteogenesis using the “Osteogenesis PCR Library”. Among these, expression of seven mRNAs was upregulated by siRNA-mediated KD of both PUM1 and PUM2 (Additional file 1: Fig. S9A), and we confirmed that the 3’-UTRs of DLX5, ITGA2, FGFR2, and TGFBR2 had PUM-binding elements (PBEs) with the sequences [UGUA(n)AUA] (Additional file 1: Table S1). Next, we used RPISeq (RNA–protein interaction prediction) to predict which of the selected osteogenesis-related mRNAs could interact with PUM2. Most of the mRNAs selected in this study scored > 0.5 and were considered to have positive interactions (Additional file 1: Fig. S9B). However, only DLX5 mRNA expression was stabilized by PUM2-KD in human MSCs (Fig. 3A). DLX5 expression was increased by PUM2-KD, whereas PUM2 overexpression decreased DLX5 levels in human MSCs (Fig. 3B, C). To confirm that PUM2 binds to DLX5 mRNA, RNA-immunoprecipitation (RNA-IP) was performed using MSCs transfected with the pEGFP-C1-PUM2 vector, and the levels of DLX5 mRNA bound with PUM2 were enriched (Fig. 3D). To investigate whether a cause-and-effect relationship exists between the decrease in PUM2 expression and increased DLX5 levels, we cultured MSCs overexpressing pEGFP-C1-tagged PUM2 or pEGFP-C1-tagged PUM2 mutant lacking the RNA-binding domain (PUM2-ΔHD). Overexpression of PUM2-ΔHD, and not PUM2, did not affect DLX5 levels in human MSCs (Fig. 3E), suggesting that the HD domain of PUM2 is essential for the PUM2-mediated decrease in DLX5 expression.

**Fig. 4:**
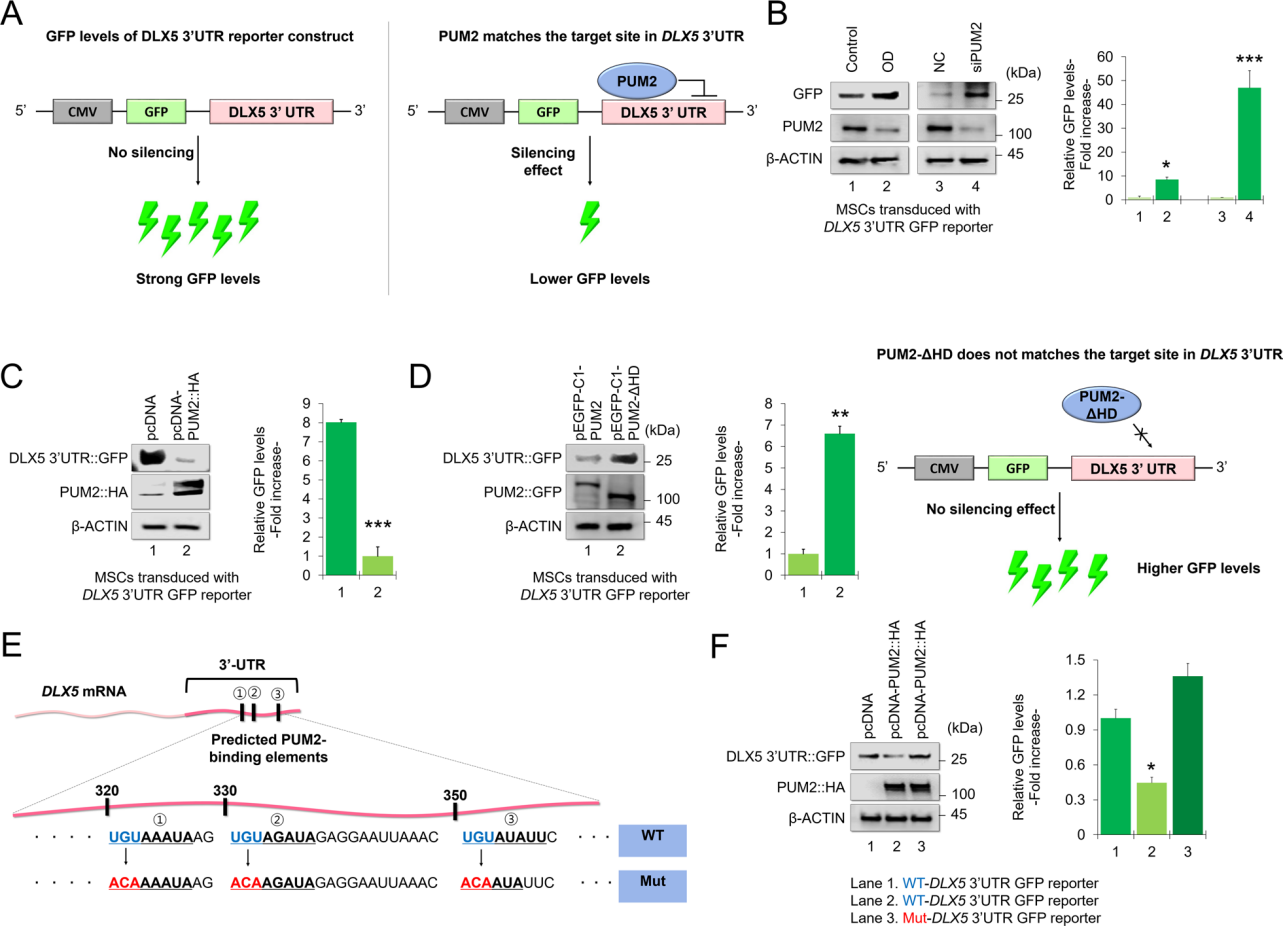
PUM2 downregulated the activity of DLX5 3’-UTR reporter in MSCs. **A** Schematic diagram of the experimental method showing the activity of human DLX5 3’-UTR downstream of a CMV-driven reporter (GFP) by PUM2. **B** Protein levels of PUM2 and GFP in MSCs transduced with DLX5 3’-UTR reporter-GFP vector. Cells were selected using puromycin (10 µg/ml) and then transfected with NC or *PUM2* siRNA before western blot to compare the GFP activities. *P < 0.05 or *P < 0.001 compared with the NC (n = 3 experimental replicates). **C,** **D** Protein levels of PUM2::HA, PUM2::GFP, and DLX5 3’UTR::GFP in MSCs transduced with the DLX5 3’-UTR reporter-GFP vector. The cells were selected using puromycin (10 µg/ml) and transfected with the pcDNA or pEGFP-C1 vector control, pcDNA-PUM2-HA, pEGFP-C1-PUM2, or pEGFP-C1-ΔPUM2-HD vector; cells were analyzed by western blot to compare the GFP activities. **P < 0.01 or ***P < 0.001 compared with the pcDNA or pEGFP-C1 vector control (n = 3 experimental replicates). **E** Schematic diagram of point mutation of the GFP/DLX5 mRNA 3’-UTR construct. To determine if PUM2 actually binds to the putative sequences [UGUA(N)AUA] within the 3’-UTR region of *DLX5* mRNA, the *DLX5* 3’-UTR GFP reporter assay was performed using mutated constructs. **F** Protein levels of PUM2::HA and DLX5 3’UTR::GFP in MSCs transduced with the wild-type DLX5 3’-UTR reporter-GFP vector or the mutant DLX5 3’-UTR reporter-GFP vector. The cells were selected using puromycin (10 µg/ml) and transfected with the pcDNA vector control or pcDNA-PUM2-HA vector; cells were analyzed by western blot to compare the GFP activities. **P < 0.01 or ***P < 0.001 compared with the pcDNA vector control (n = 3 experimental replicates)

To further understand whether PUM2 interacts with the DLX5 3’-UTR containing PBEs, we transduced human MSCs with a lentiviral vector including human DLX5 3’-UTR downstream of a CMV-driven reporter (green fluorescent protein [GFP]) (Fig. 4A). We then transfected the cells with siRNAs (NC or PUM2 siRNA) or expression vectors (pcDNA3-mock, pcDNA3-PUM2::HA, pEGFP-C1-PUM2, or pEGFP-C1-ΔPUM2-HD) under osteogenic conditions. The relative activity of the DLX5 3’UTR-GFP reporter was enhanced in MSCs transfected with siRNA targeting PUM2 and cultured in osteogenic medium (Fig. 4B). Conversely, reporter activity was reduced in cells transfected with the pcDNA-PUM2 expression vector under osteogenic conditions (Fig. 4C), whereas transfection with the pEGFP-C1-PUM2-ΔHD vector enhanced the GFP levels of reporter compared to the pEGFP-C1-PUM2 wild-type vector (Fig. 4D). These data suggest that PUM2-KD-mediated enhancement of MSC osteogenesis was regulated by preventing the negative regulation of the DLX5 3’-UTR by PUM2. Next, we mutated three regions where PUM2 is expected to bind to the 3'-UTR of *DLX5* mRNA. PUM2 can bind sequence-specifically to an RNA sequence called UGUA(n)AUA. Therefore, the UGUA(n)AUA part of the existing DLX5 3'-UTR-GFP vector was point-mutated into ACAA(n)AUA (Fig. 4E, and Additional file 1: Fig. S2). We then performed a GFP reporter assay using both the original wild-type and mutant vectors to determine the PUM2-binding site within the DLX5 3’-UTR region. The results showed that PUM2 overexpression reduced the GFP level in the wild-type DLX5 3’-UTR GFP reporter, whereas PUM2 overexpression did not show any reducing effect on the GFP level in MSCs transduced with the mutant DLX5 3’-UTR GFP reporter (Fig. 4F). Overall, these results suggest that PUM2 inhibits translation of DLX5 by binding to the pumilio-binding motif (UGAU(n)AUA) present in the 3'-UTR of DLX5 mRNA using its own HD domain.

### *AAV9-mediatedsilencingofPUM2mitigatesOVX-inducedboneloss*

**Fig. 5:**
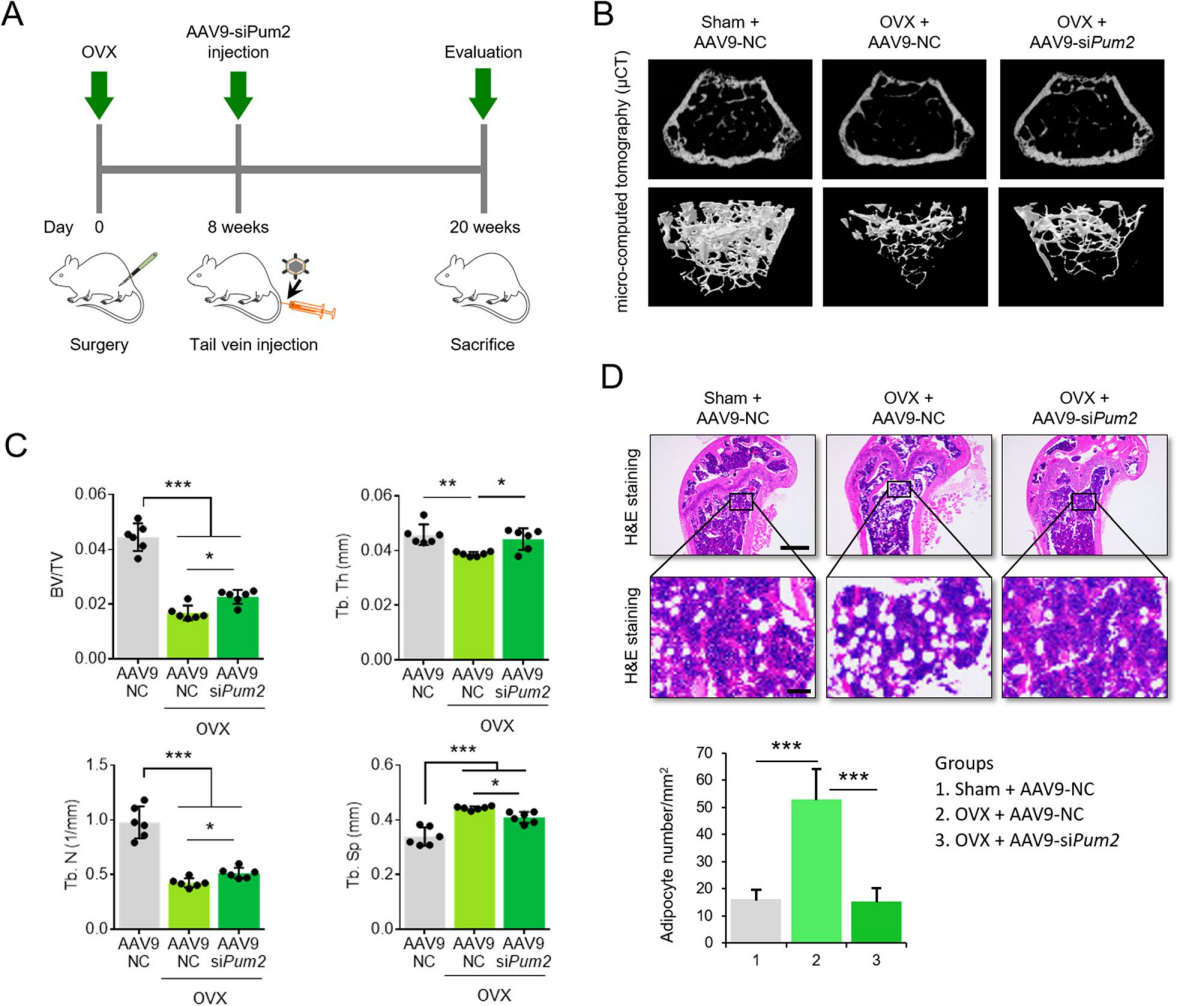
AAV9-mediated gene silencing of *Pum2* alleviates the osteoporotic phenotypes in OVX-induced mice. **A** Schematic of the time course used for the in vivo experiments in mice with OVX-induced osteoporotic phenotypes. Intravenous injection of 200 μL each of AAV9-NC (approximately 2.5 × 10^10^ GC/mL) or AAV9-si*Pum2* (approximately 2.6 × 10^10^ GC/mlL was administered to commercially available female OVX mice or sham-operated control mice of the same age. Twelve weeks after surgery, the mice were sacrificed for further experiments. **B** Femoral samples from each group were collected and subjected to μCT analysis to examine osteoporotic phenotypes. Representative μCT images of fracture calluses at twelve weeks post AAV9 injection. μCT showed that systemic injection of AAV9-si*Pum2* could alleviate bone loss. **C** The bar graphs shows quantitative analysis of bone-related parameters, including BV/TV, Tb.Th (mm^2^), Tb.N (1/mm^2^), and Tb. Sp (mm^2^) (n = 6). The data are expressed as the mean ± SD; *P < 0.05, **P < 0.01, and ***P < 0.001. BV/TV, bone volume per tissue volume; Tb.Th, trabecular thickness; Tb.N, trabecular number; Tb. Sp, trabecular spacing. **D** Histological analysis of bone loss and lipid accumulation in the femur was performed using hematoxylin and eosin staining. Scale bar = 1 mm (upper) or 50 μm (lower). Accumulation of lipid droplets in the bone marrow was quantified by counting droplets per mm^2^ (experimental triplicate, n = 3). The data are expressed as the mean ± SD; ***p < 0.001

RNAi-based gene therapy is a promising tool for preventing bone loss during osteoporosis [32, 33]. Adeno-associated virus (AAV)-mediated gene therapy has been suggested as a vehicle for bone tissue-specific gene silencing [34]. Among the various serotypes of AAV, AAV9 is the most efficient for gene delivery into bone tissues [35, 36]. To investigate whether *PUM2* silencing is effective in preventing bone loss during osteoporosis, AAV9-si*Pum2* was used in a study of ovariectomized (OVX) mice. We administered tail vein injections of AAV9-negative control (NC) siRNA or AAV9-si*Pum2* into 8-week-old commercially available OVX mice. Twelve weeks later, the mice were sacrificed for evaluation (Fig. 5A). μCT showed the morphologies of the tibia and femur, and representative 2- and 3-dimensional images in the OVX group injected with AAV9-NC showed high trabecular bone loss compared with those of the sham injected with AAV9-NC, whereas the group injected with AAV9-si*Pum2* showed less trabecular bone loss (Fig. 5B). Furthermore, bone volume/tissue volume (BV/TV) ratio, trabecular thickness (Tb. Th), and trabecular number (Tb. N) were higher in the OVX AAV9-si*Pum2* group than in the OVX AAV9-NC group. The trabecular separations (Tb. Sp) were slightly lower in the OVX AAV9-si*Pum2* group than in the OVX AAV9-NC group (Fig. 5C). Increased bone marrow adiposity is an osteoporotic bone marker [37, 38]. To determine whether injection of the AAV9-si*Pum2* affected bone marrow adiposity in OVX mice, marrow adipogenesis was measured histologically in harvested tibiae. Hematoxylin and eosin staining showed that the number of adipocytes increased in OVX mice injected with AAV9-NC compared to sham mice injected with AAV9-NC. Injection of AAV9-si*Pum2* reduced adiposity in OVX mice (Fig. 5D). These results suggest that *PUM2* may be a potential target for osteoporosis gene therapy.

## Discussion

PUMILIO proteins are sequence-specific RBPs, and their functions are well-conserved between species, including *Drosophila* and humans [39–41]. Mammals have two classical PUMILIO proteins, PUM1 and PUM2, which have a conserved RNA-binding domain (PUM-HD) [39]. PUM1 and PUM2 bind to the 3’-UTRs of mRNAs containing PUMILIO response elements (PREs; UGUA_AUA), and PUMILIO binding stimulates the degradation of target mRNAs or inhibits translation [42]. PUM1 and PUM2 are involved in various biological processes and regulatory functions in mammals [39]. Dysfunction of PUM1 and PUM2 not only results in embryonic lethality and developmental disability [43, 44] but also in reduced fertility caused by *PUM1* and *PUM2* knockout [45–47]. Defects in proliferation and differentiation capacity of embryonic [48], germline [45], hematopoietic [49, 50], and neural stem cells [43] in humans and mice are observed when PUM1 or PUM2 are knocked down or knocked out. PUMILIO is involved in the proliferation and differentiation of cells of various lineages, but there are few studies on adult progenitor cells that can differentiate into mesenchymal lineages. Shigunov et al. reported that PUM2 could positively regulate the proliferative capacity of adipose-derived MSCs but did not play a significant role in their differentiation capacity [51]. However, human bone marrow-derived MSCs failed to differentiate into osteoblasts following overexpression of *PUM2* [15]. We showed that PUM1 and PUM2 may act differently, and that loss of PUM1 rather than PUM2 may affect the senescence and chondrogenic differentiation capacity of bone marrow-derived MSCs [21]. Several studies have focused on elucidating the role of PUM2 in MSCs, but the molecular mechanism of PUM2 in MSC differentiation and its clinical applications remain unknown. Here, we demonstrated that PUM2 KD could enhance the osteogenic potential of MSCs, and PUM2 KD increased the efficacy of MSC-driven bone regeneration in a rat calvarial defect model, suggesting that *PUM2*-deficient MSCs may be an effective cell therapy for bone regeneration. Additionally, we confirmed that osteoporosis progression could be partly controlled by injecting AAV9-si*Pum2*, indicating a potential gene therapy agent to control *PUM2* expression in vivo.

We showed that PUM2 negatively regulates DLX5 expression by binding to its 3’-UTR. DLX5 is a master regulator of MSC osteogenesis [52], and DLX5 expression during osteogenesis triggers the expression of other important regulators, such as RUNX2, Osteopontin, and ALP, according to the sequential pathway during skull development [53]. RUNX2 expression downstream of BMP2 provides key signaling for MSC differentiation into osteoblasts. During BMP2 treatment, DLX5 binds to the *RUNX2* distal promoter to regulate its expression [10, 54]. Previously, we showed DLX5 is involved in the osteogenic lineage commitment of human MSCs [55] and regulates *STAT5A* deletion-mediated stimulation of bone remodeling in the fracture callus [56]. Although increased expression of DLX5 in MSCs is a key factor in differentiation into osteoblasts, little is known about how DLX5 is regulated post-transcriptionally. DLX5 is important for maintaining bone homeostasis and skeletal integrity [57], and understanding its various regulatory mechanisms will enhance knowledge in the field of osteobiology. Here, we discovered *DLX5* is a PUM2 target mRNA during MSC osteogenesis. Although more target mRNAs of PUM2 may be present in MSCs, and they may also be involved in various regulatory pathways, we showed for the first time how DLX5 is post-transcriptionally activated as a target of the RBP PUM2 during MSC osteogenesis.

The current study could not provide precise guidelines and protocols for the regeneration of damaged bone tissue and the treatment of osteoporosis using PUM2-KD MSCs or AAV9-si*Pum2*. If various gene-modifying technologies that can effectively delete PUM2 from MSCs, such as CRISPR/CAS9 technology [58] or advanced antisense oligonucleotide technology [59, 60], are employed, PUM2 could be more stably applied as a cell therapy target for bone regeneration. Another shortcoming of this study is that systemic delivery of *Pum2* siRNA using the AAV9 vector was performed only once in OVX mice. Even with a single injection, we obtained results that could partially control OVX-induced bone loss. Although AAV9 is the current most effective AAV serotype capable of targeting bone tissue [36], better results are expected if the number of injections or safety of the treatment becomes clear in the future. It would also be best if we could construct bone tissue-specific *Pum2* conditional knockout mice and elucidate the role of PUM2 during osteogenesis in vivo. However, at this point, the focus of current study was to evaluate whether regulation of *PUM2* expression would be of value as a candidate gene for bone tissue regeneration using stem cells or for osteoporosis gene therapy. These limitations will be supplemented through future research. Nevertheless, the approaches in the current study will advance the future development of stem cell therapies for bone regeneration or gene therapies for osteoporosis.

## Conclusions

**Fig. 6:**
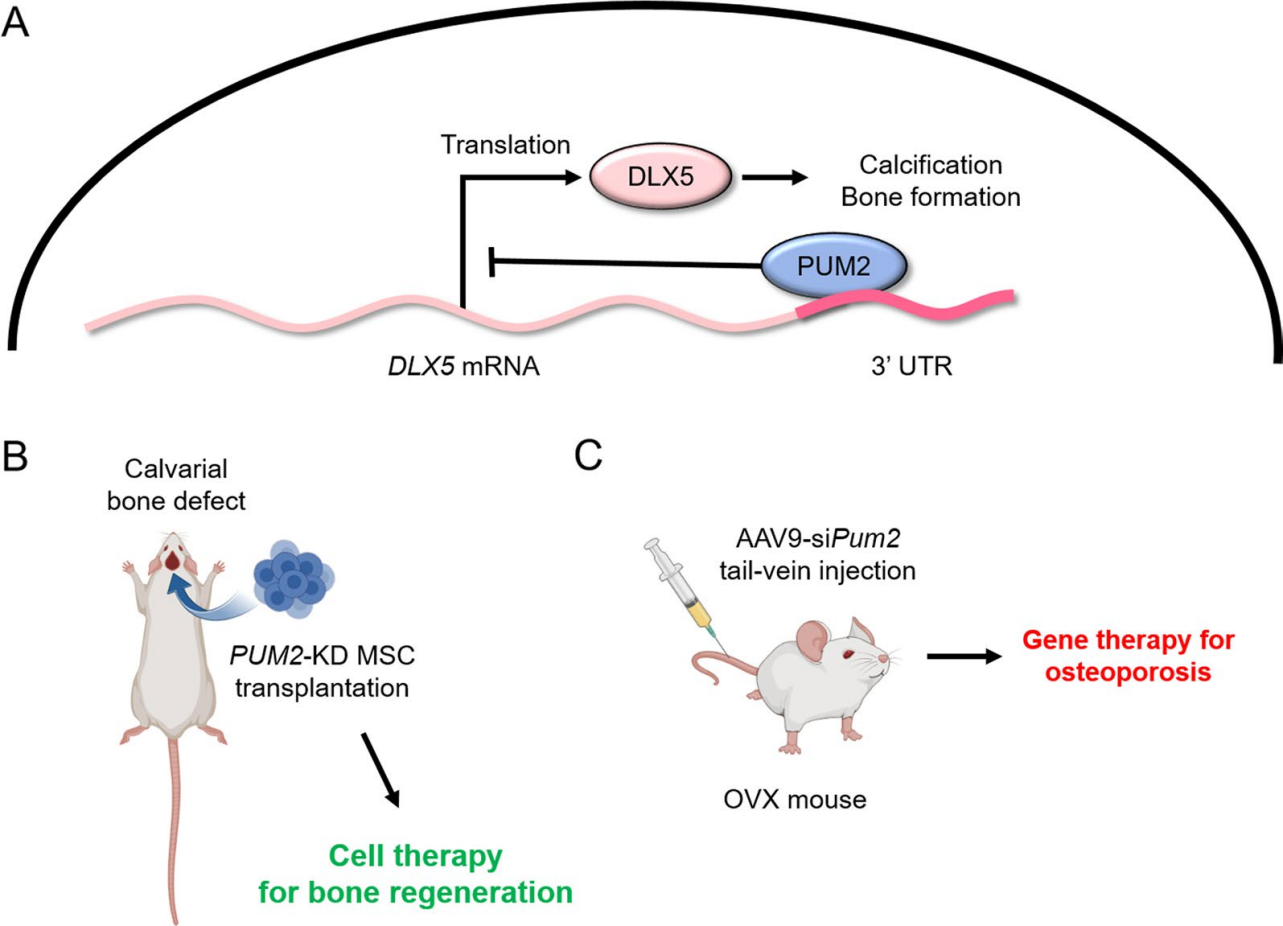
Graphical abstract of PUM2-mediated osteogenesis in MSCs. **A** PUM2 inhibits translation of DLX5 by interacting with the 3’-UTR of DLX5 mRNA. **B** MSCs with PUM2 knockdown showed potential as cell therapy for the regeneration of damaged bone tissue. **C** AAV9 loaded with siRNA targeting Pum2 showed potential as a gene therapy that can alleviate osteoporosis symptoms through systemic injection

In summary, our findings contribute to the understanding of the mechanisms that regulate an increased level of DLX5 post-transcriptionally in MSCs during osteogenic differentiation (Fig. 6A). Downregulation of PUM2 expression committed MSCs into the osteogenic lineage, leading to enhanced osteogenic potential under both in vitro and in vivo conditions (Fig. 6B). In addition, the systemic delivery of PUM2 siRNA prevented OVX-induced bone loss (Fig. 6C). Therefore, we suggest that PUM2-mediated DLX5 regulation may be an important regulatory axis for osteogenic differentiation of human MSCs, and PUM2 could be a target for gene therapy to slow or prevent osteoporosis progression by modulating DLX5-mediated osteogenesis of MSCs.

## Supplementary Information

Additional file 1: Method. Flow cytometry, Isolation and cultivation of rat andmouse bone marrow-derived MSCs, RNA sequencing analysis. Fig. S1. Preparation of PUM2 mutant lacking the RNA-binding domain. Fig. S2. Point mutation of the GFP/DLX5 mRNA 3’-UTR construct at 319 to 321 nt, 328 to 331 nt, and 348 to 351 nt. Fig. S3. In vivo fluorescence imaging to monitor AAV9 injection. Fig. S4. Knockdown of PUM2 in MSCs increases the ALP-positive cell population. Fig. S5. The effect of fibrin glue gel on the self-regeneration ability of rats with calvarial defects. Fig. S6. Detection of immune cells in transplanted sites of xenograft human MSCs by immunohistochemistry. Fig. S7. Western blot analysis using a human-specific vimentin antibody. Fig. S8. Effect of Pum2 knockdown in MSCs isolated from rat bone marrow. Fig. S9. Analysis to find potential RNA targets that can interact with PUM2 during MSC osteogenesis. Table S1. List of 3’-UTRs for human DLX5, FGFR3, EGF, MSX1, ITGA2, FGFR2, and TGFBR2. Red-colored letters mean PBEs with the exact sequences, and blue-colored letters mean PBEs with possible binding motifs. Table S2. List of primers used in the present study. Table S3. siRNAs used in the current study.

## Data Availability

The data presented in current study are included in the article and supplemental material.

## Abbreviations

AAV9: Adeno-associated virus serotype 9
ALP: Alkaline phosphatase
ANOVA: Analysis of variance
BV/TV: Bone volume/tissue volume
CMV: Cytomegalovirus
COL1A1: Collagen type I alpha 1 chain
copGFP: Copepod green fluorescent protein
DLX5: Distal-Less Homeobox 5
DMEM-LG: Dulbecco’s modified eagle’s low-glucose medium
FACS: Fluorescence-activated cell sorting
FBS: Fetal bovine serum
FGFR2: Fibroblast growth factor receptor 2
GFP: Green fluorescent protein
HEK293: Human embryonic kidney 293
IHC: Immunohistochemistry
IP: Immunoprecipitation
ITGA2: Integrin subunit alpha 2
KD: Knockdown
MSCs: Mesenchymal stem cells
NC: Negative control
OCN: Osteocalcin
OPN: Osteopontin
OVX: Ovariectomized
PBEs: PUMILIO-binding elements
PBS: Phosphate-buffered saline
PUM1: PUMILIO1
PUM2: PUMILIO2
PUM-HD: PUMILIO homology domain
PREs: PUMILIO response elements
qPCR: Quantitative polymerase chain reaction
RBPs: RNA-binding proteins
RNA-IP: RNA-immunoprecipitation
RPISeq: RNA–protein interaction prediction
RUNX2: Runt related transcription factor 2
PPARγ: Peroxisome proliferator-activated receptor gamma
SDS: Sodium dodecyl sulfate
siRNA: Small interfering RNA
Tb. N: Trabecular number
Tb. Sp: Trabecular separation
Tb. Th: Trabecular thickness
TGFBR2: Transforming growth factor beta receptor 2
UTRs: Untranslated regions
Δ: Deletion
µCT: X-ray micro-computed tomography
