# Supplementary material for: Downregulation of the RNA-binding protein PUM2 facilitates MSC-driven bone regeneration and prevents OVX-induced bone loss
Source: J Biomed Sci. 2023 Apr 23;30:26. doi: 10.1186/s12929-023-00920-8 (PMC10122812; doi:10.1186/s12929-023-00920-8)
Supplement: Supplementary file 1 — Additional file 1: Method. Flow cytometry, Isolation and cultivation of rat andmouse bone marrow-derived MSCs, RNA sequencing analysis. Fig. S1. Preparation of PUM2 mutant lacking the RNA-binding domain. Fig. S2. Point mutation of the GFP/DLX5 mRNA 3’-UTR construct at 319 to 321 nt, 328 to 331 nt, and 348 to 351 nt. Fig. S3. In vivo fluorescence imaging to monitor AAV9 injection. Fig. S4. Knockdown of PUM2 in MSCs increases the ALP-positive cell population. Fig. S5. The effect of fibrin glue gel on the self-regeneration ability of rats with calvarial defects. Fig. S6. Detection of immune cells in transplanted sites of xenograft human MSCs by immunohistochemistry. Fig. S7. Western blot analysis using a human-specific vimentin antibody. Fig. S8. Effect of Pum2 knockdown in MSCs isolated from rat bone marrow. Fig. S9. Analysis to find potential RNA targets that can interact with PUM2 during MSC osteogenesis. Table S1. List of 3’-UTRs for human DLX5, FGFR3, EGF, MSX1, ITGA2, FGFR2, and TGFBR2. Red-colored letters mean PBEs with the exact sequences, and blue-colored letters mean PBEs with possible binding motifs. Table S2. List of primers used in the present study. Table S3. siRNAs used in the current study. [file 12929_2023_920_MOESM1_ESM.docx]

**Additional file 1**

**Method**: Flow cytometry, Isolation and cultivation of rat and mouse bone marrow-derived MSCs, RNA sequencing analysis

**Fig. S1. Preparation of PUM2 mutant lacking the RNA-binding domain (PUM2-ΔHD).**

**Fig. S2. Point mutation of the GFP/DLX5 mRNA 3’-UTR construct at 319 to 321 nt (mutant site 1), 328 to 331 nt (mutant site 2), and 348 to 351 nt (mutant site 3).**

**Fig. S3. *In vivo* fluorescence imaging to monitor AAV9 injection.**

**Fig. S4. Knockdown of *PUM2* in MSCs increases the ALP-positive cell population.**

**Fig. S5. The effect of fibrin glue gel on the self-regeneration ability of rats with calvarial defects.**

**Fig. S6. Detection of immune cells in transplanted sites of xenograft human MSCs by immunohistochemistry.**

**Fig. S7. Western blot analysis using a human-specific vimentin antibody.**

**Fig. S8. Effect of *Pum2* knockdown in MSCs isolated from rat bone marrow.**

**Fig. S9. Analysis to find potential RNA targets that can interact with PUM2 during MSC osteogenesis.**

**Table S1** List of 3’-UTRs for human *DLX5*, *FGFR3*, *EGF*, *MSX1*, *ITGA2*, *FGFR2*, and *TGFBR2*. Red-colored letters mean PBEs with the exact sequences, and blue-colored letters mean PBEs with possible binding motifs. (Source: <https://genome.ucsc.edu/>)

**Table S2** List of primers used in the present study.

**Table S3** siRNAs used in the current study.**Method:**

***Flow cytometry***

NC or *PUM2* siRNA-transfected MSCs (1 × 10^6^) were stained with CD34-FITC (Miltenyi Biotec, Auburn CA, USA), FITC-mouse-IgG2a isotype control (Miltenyi Biotec), CD45-PerCP (Miltenyi Biotec), PerCP-mouse IgG2a isotype control (Miltenyi Biotec), CD90-APC (Miltenyi Biotec), APC-mouse IgG1 isotype control (Miltenyi Biotec), CD105-PE-Cy7 (BioLegend, San Diego, CA, USA), PE-Cy7-mouse IgG1 isotype control (BD Biosciences), and W8B2 (antibody against MSCA-1)-APC (Miltenyi Biotec) for 30 min at 4°C. Thereafter, the stained cells were washed with phosphate-buffered saline (PBS), centrifuged at 300 × g for 10 min, and resuspended in 500 mL of ice-cold PBS supplemented with 1% FBS and 0.05% sodium azide. The samples were subjected to FACS analysis using a FACSverse flow cytometer (BD Biosciences). The samples were analyzed using FlowJo v10 software (BD Biosciences), with an unstained sample (1 × 10^6^) as a negative control.

***Isolation and cultivation of rat and mouse bone marrow-derived MSCs***

Twelve-week-old male Sprague-Dawley rats and six-week-old male mice were used for isolation of bone marrow-derived MSCs. Femurs and tibias were isolated from rat or mice by flushing the bone marrow with a 27-gauge needle and filtered through a 70-μm cell strainer (Falcon, Tewksbury, MA, USA). The isolated cells were centrifuged at 1,500 rpm for 10 min and resuspended in low-glucose Dulbecco’s modified Eagle’s medium (DMEM-LG; Invitrogen, Carlsbad, CA, USA) supplemented with 10% fetal bovine serum (FBS; Gibco, Grand Island, NY, USA) and 1% antibiotic–antimycotic solution (Invitrogen) at 37°C and 5% CO2 and were subcultured at 80% confluency. To induce MSC differentiation into the osteogenic lineage, the rat or mouse MSCs were seeded at 8 × 10^4^ cells/well in 12-well plates and cultured in osteogenic medium [DMEM-LG containing 10% FBS, 1% antibiotic-antimycotic solution, 10 mM β-glycerophosphate (Sigma-Aldrich, St. Louis, MO, USA), and 50 μg/mL ascorbic acid (Gibco)] for 10 days. The medium was replaced every 2 days. Alizarin red S staining was performed to determine the osteogenic capacity of MSCs. After fixation in ice-cold 70% ethanol, 1 mL of freshly prepared 3% alizarin red S solution (wt/vol) (Sigma-Aldrich) was added before incubation in the dark for 30 min.

***RNA sequencing analysis***

From Bohn et al. (See Reference 31 in the main text of the manuscript), we identified potential target mRNAs of PUM2 during MSC osteogenesis using the “Osteogenesis PCR Library” in the qPCR array library from Bioneer (<https://www.bioneer.co.kr/literatures/pdf/qPCR_Array_Library_file/Cellular_DifferentiationNRegulation/Osteogenesis_PCR_Library.pdf>), and this list is available for anyone to use. Expression changes of related genes involved in the osteogenesis process of human MSCs were confirmed using this well-arranged library. RPISeq (RNA-protein interaction prediction) is an algorithm to predict the possible RNAs that could interact with a protein. We used RPISeq to analyze PUM2 and the selected osteogenesis-related mRNAs determined above ([*http://pridb.gdcb.iastate.edu/RPISeq/about.php#basic*](http://pridb.gdcb.iastate.edu/RPISeq/about.php#basic)).

**
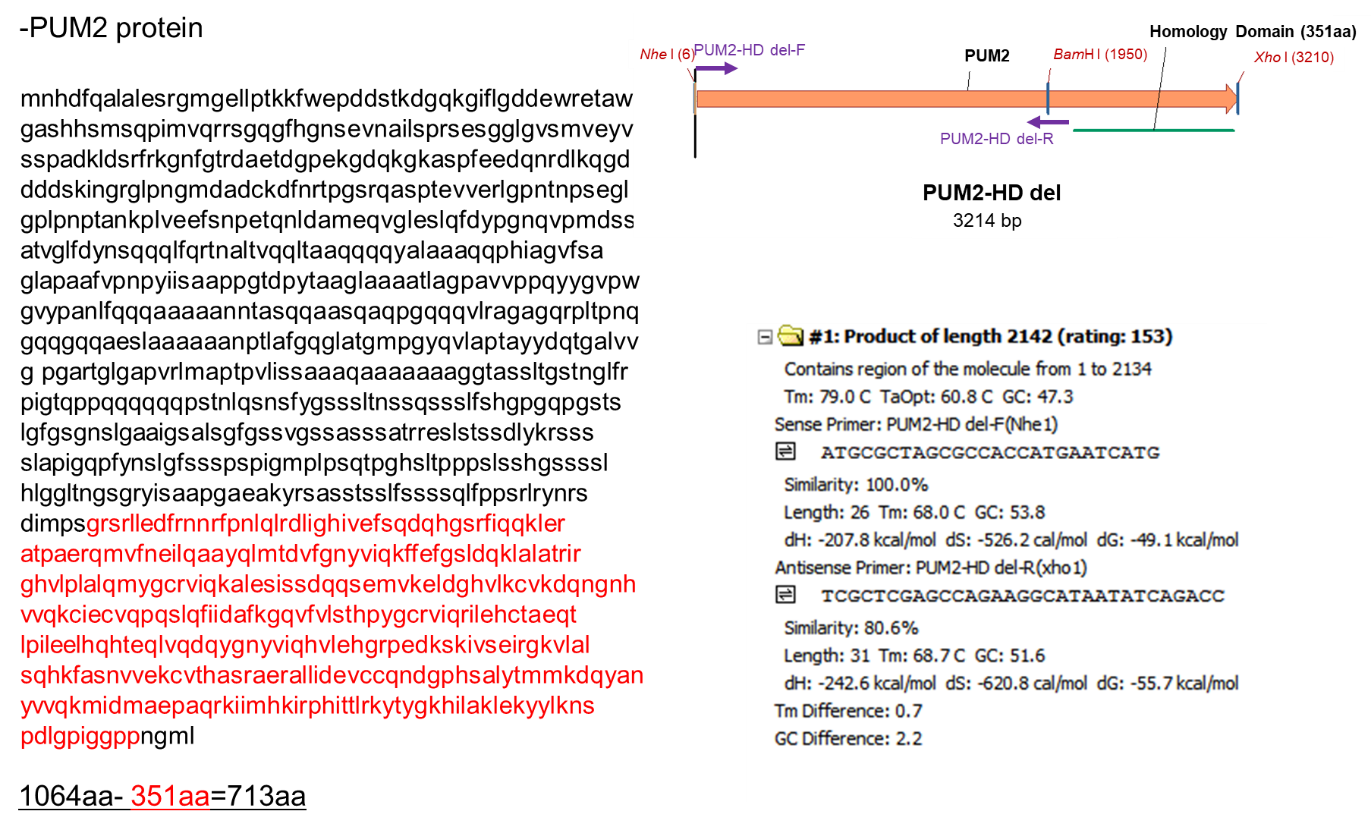
**

**Fig. S1. Preparation of PUM2 mutant lacking the RNA-binding domain (PUM2-ΔHD).** ΔPUM2-HD was cloned into the pEGFP-C1 vector between the NheI and XhoI sites to generate pEGFPC1-ΔPUM2-HD that expresses a green fluorescent protein (GFP)-PUM1 fusion protein. The red region indicates the part deleted for PUM2-ΔHD vector generation.


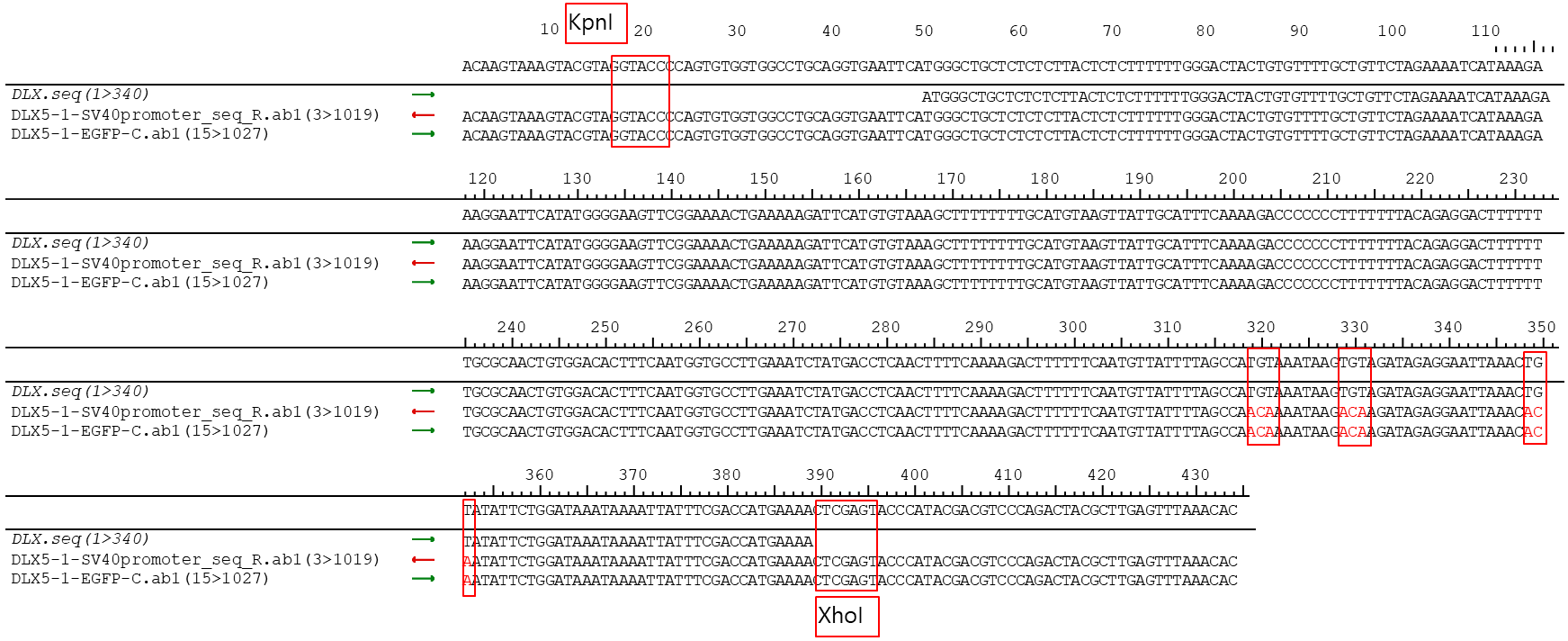


**Fig. S2. Point mutation of the GFP/DLX5 mRNA 3’-UTR construct at 319 to 321 nt (mutant site 1), 328 to 331 nt (mutant site 2), and 348 to 351 nt (mutant site 3).** To determine if PUM2 actually binds to the putative sequences [UGUA(N)AUA] within the 3’-UTR region of *DLX5* mRNA, the *DLX5* 3’-UTR GFP reporter assay was performed using mutated constructs, and the mutated plasmid was confirmed by sequencing.

**
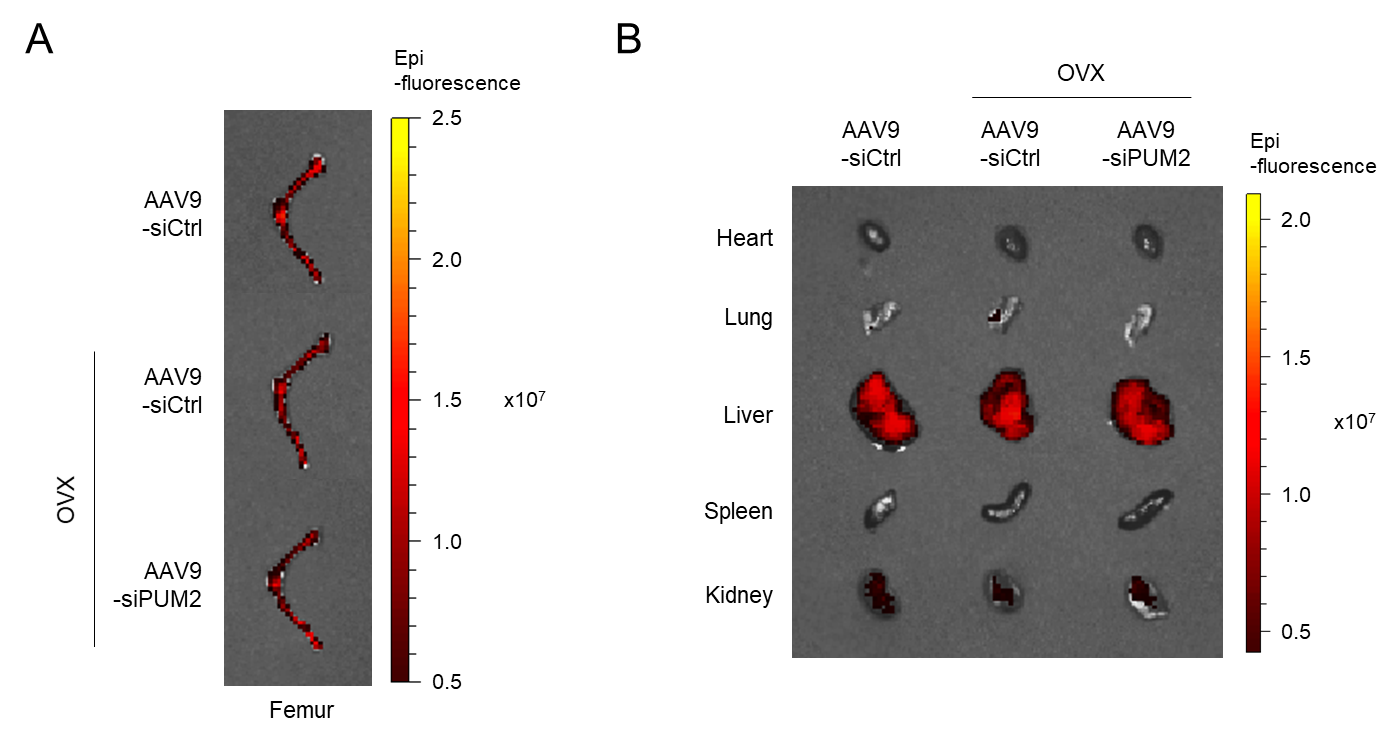
**

**Fig. S3. *In vivo* fluorescence imaging to monitor AAV9 injection. A, B** A single dose of 5 × 10^9^ genome copies of each AAV9-GFP was intravenously injected into 8-weeks-old female sham or OVX mice, and GFP expression in the hindlimb and individual tissues was monitored by IVIS-100 optical imaging 12 weeks post injection. *y*-axis, radiant efficiency (p/s/cm^2^/sr/μW/cm^2^).

**
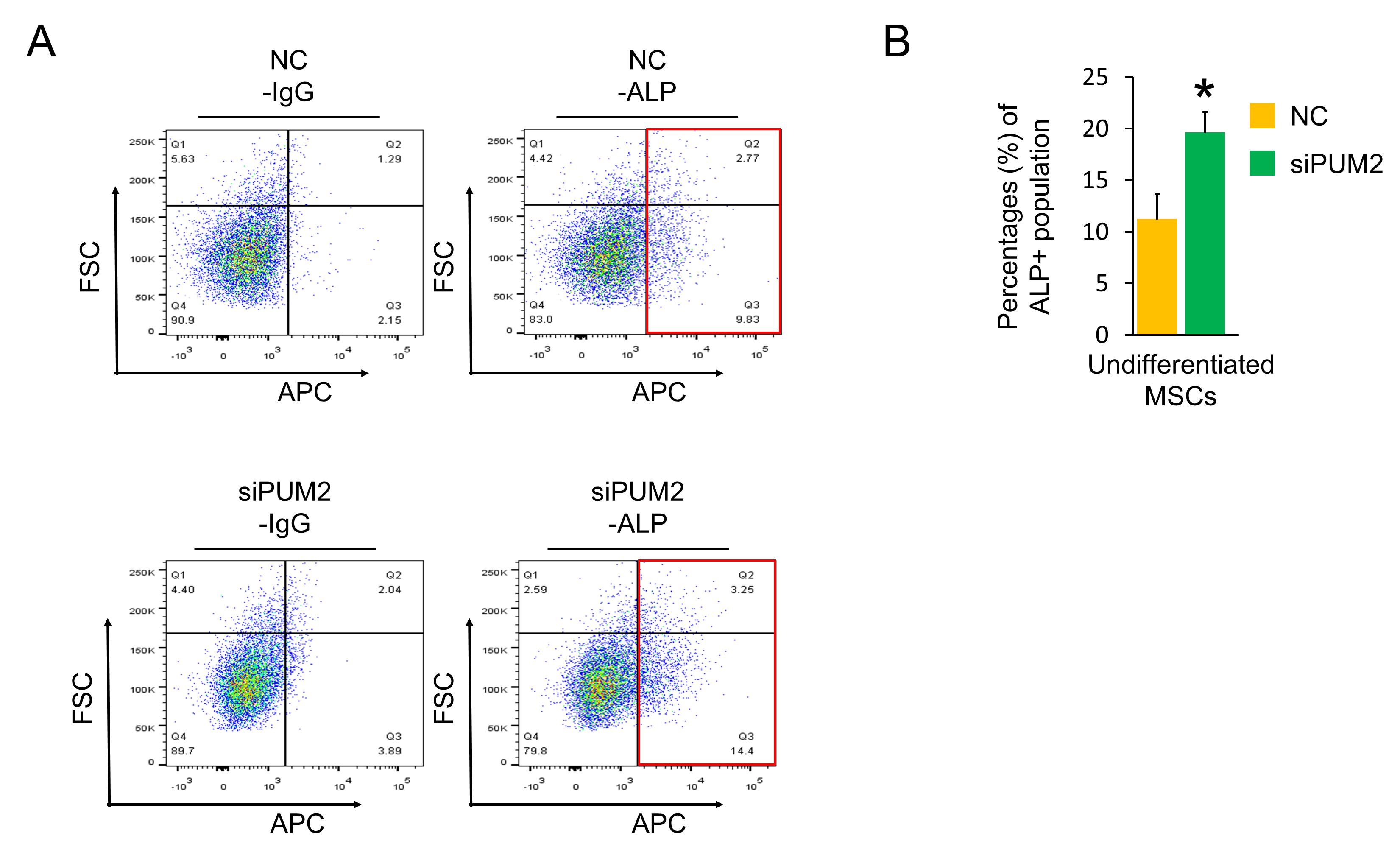
**

**Fig. S4. Knockdown of *PUM2* in MSCs increases the ALP-positive cell population. A** NC or *PUM2* siRNA-transfected MSCs were immunostained with an antibody against tissue non-specific ALP. The stained cells were analyzed using flow cytometry to detect the surface markers specific for MSCs (n = 3 experimental replicates). **B** The graph shows the percentages of ALP-positive cell population (n = 3 experimental replicates). **P>*0.05 compared with scrambled.

**
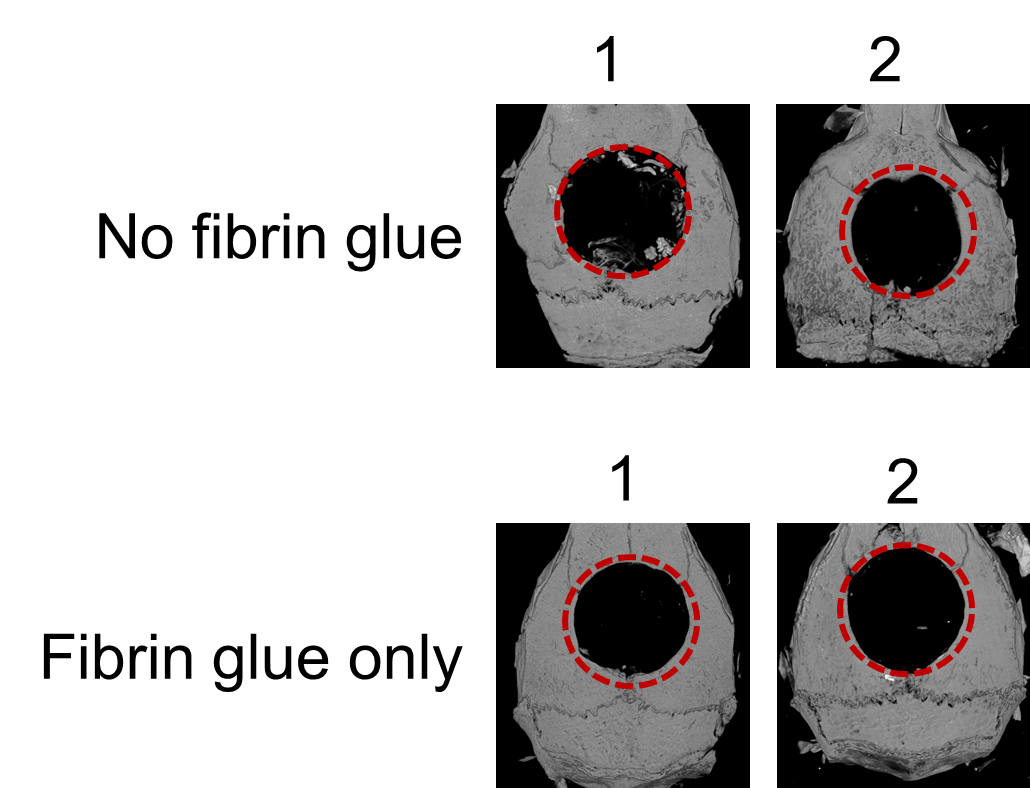
**

**Fig. S5. The effect of fibrin glue gel on the self-regeneration ability of rats with calvarial defects.** µCT images show bone regeneration at the calvarial defect at 8 weeks in the groups with no fibrin glue or only fibrin glue implanted. Fibrin gel alone was unable to self-regenerate rat calvarial defects.


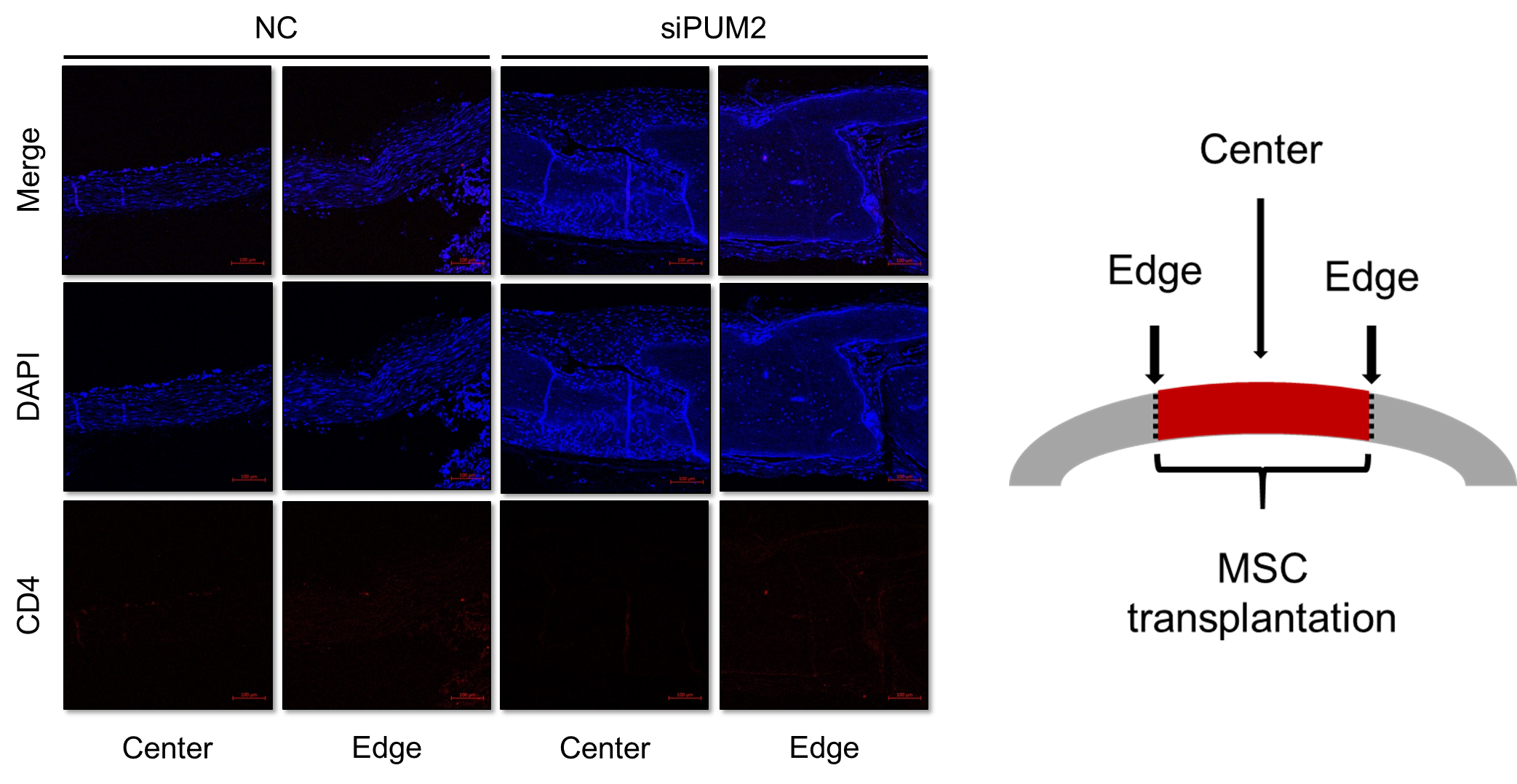


**Fig. S6. Detection of immune cells in transplanted sites of xenograft human MSCs by immunohistochemistry.** The CD4 antibody has been used to detect some types of immune cells such as T cells. Almost no immune cells were detected in the calvarial defect of rats transplanted with human MSCs.


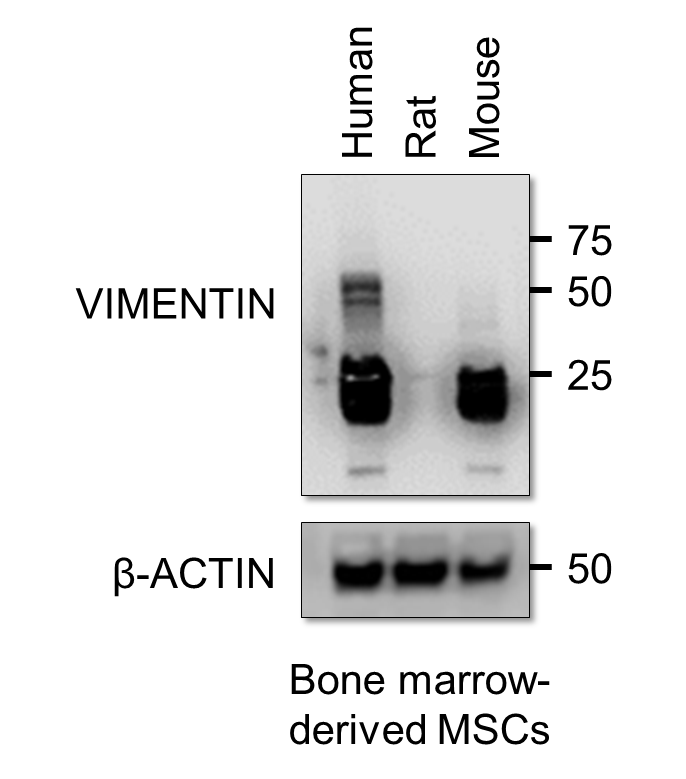


**Fig. S7. Western blot analysis using a human-specific vimentin antibody.** After extracting proteins from bone marrow-derived MSCs of humans, rats and mice, it was confirmed by western blotting whether the human-specific antibodies used in this study can detect vimentin proteins of other species.


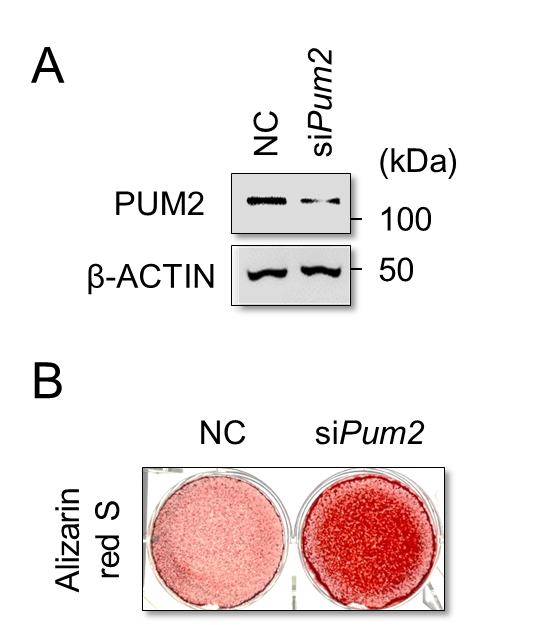


**Fig. S8. Effect of *Pum2* knockdown in MSCs isolated from rat bone marrow. A** Representative images of western blot analysis for protein level of PUM2 in in NC or rat *Pum2* siRNA-transfected MSCs. β-ACTIN was used as a loading control. **B** Representative images of alizarin red S staining in NC or rat *Pum2* siRNA-transfected MSCs cultured with ODM for 10 days.


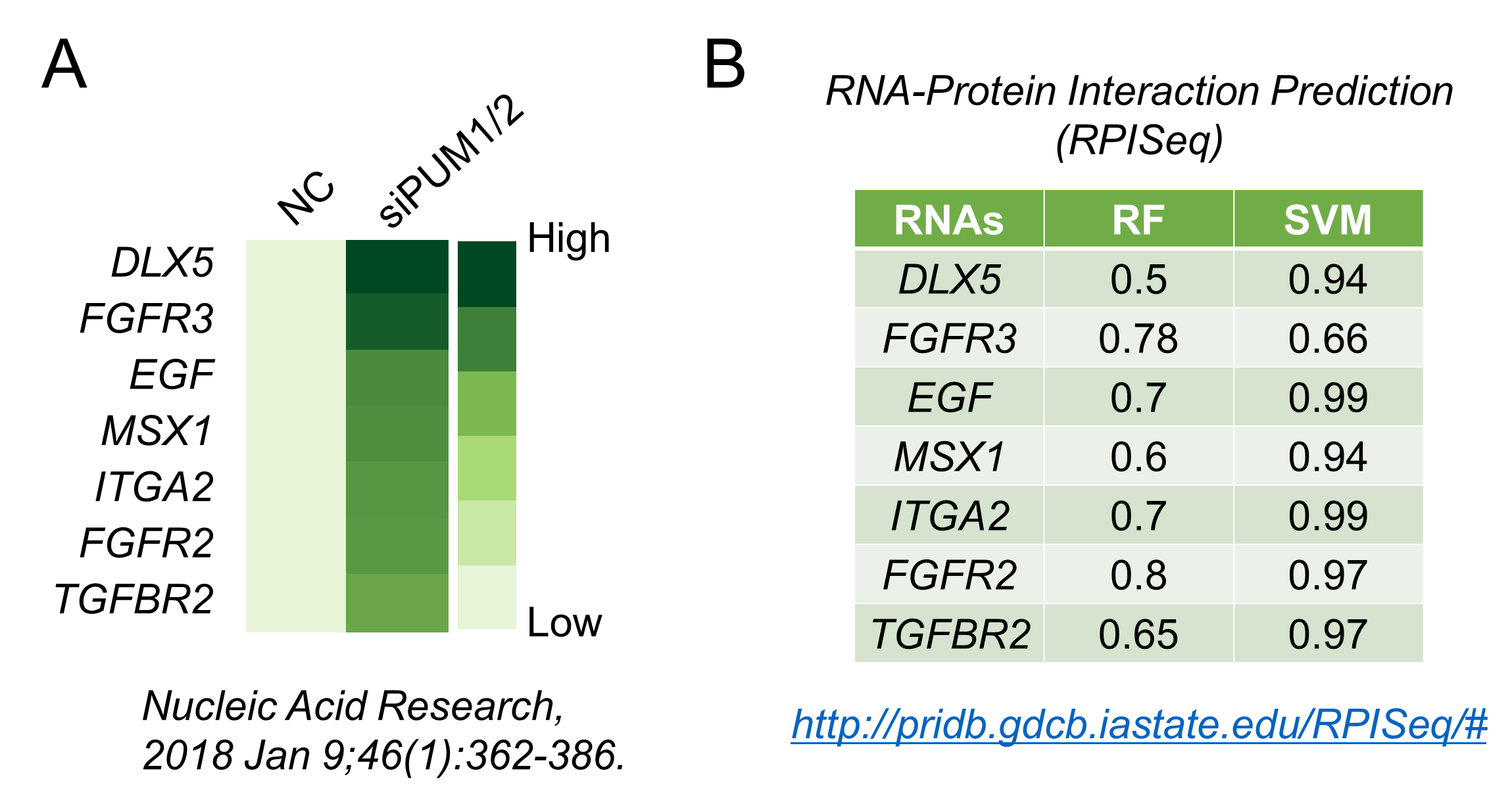


**Fig. S9. Analysis to find potential RNA targets that can interact with PUM2 during MSC osteogenesis. A** Expression of osteogenic differentiation-related genes in *PUM1*- and *PUM2*-KD cells referencing RNA-seq data from Bohn et al. (Nucleic Acids Res. 2018; Ref. 20). Results were ranked by log2-fold change. **B** RPISeq analysis predicted the interaction probability of upregulated osteogenic differentiation-related genes with PUM2. RF and SVM >0.5 was considered to have binding abilities.

**Table S1. List of 3’-UTRs for human *DLX5*, *FGFR3*, *EGF*, *MSX1*, *ITGA2*, *FGFR2*, and *TGFBR2*. Red-colored letters mean PBEs with the exact sequences, and blue-colored letters mean PBEs with possible binding motifs. (Source:** [**https://genome.ucsc.edu/**](https://genome.ucsc.edu/)**)**

| Possible target mRNA of PUM2 | 3’-UTRs | No. of PBE  (Possible binding motifs) |
| --- | --- | --- |
| DLX5 | AUGGGCUGCUCUCUCUUACUCUCUUUUUUGGGACUACUGUGUUUUGCUGUUCUAGAAAAUCAUAAAGAAAGGAAUUCAUAUGGGGAAGUUCGGAAAACUGAAAAAGAUUCAUGUGUAAAGCUUUUUUUUGCAUGUAAGUUAUUGCAUUUCAAAAGACCCCCCCUUUUUUUACAGAGGACUUUUUUUGCGCAACUGUGGACACUUUCAAUGGUGCCUUGAAAUCUAUGACCUCAACUUUUCAAAAGACUUUUUUCAAUGUUAUUUUAGCCA**UGUAAAUA**AG**UGUAGAUA**GAGGAAUUAAACUGUAUAUUCUGGAUAAAUAAAAUUAUUUCGACCAUGAAAA | 2 (1) |
| FGFR3 | AGGGCCACUGGUCCCCAACAAUGUGAGGGGUCCCUAGCAGCCCACCCUGCUGCUGGUGCACAGCCACUCCCCGGCAUGAGACUCAGUGCAGAUGGAGAGACAGCUACACAGAGCUUUGGUCUGUGUGUGUGUGUGUGCGUGUGUGUGUGUGUGUGUGCACAUCCGCGUGUGCCUGUGUGCGUGCGCAUCUUGCCUCCAGGUGCAGAGGUACCCUGGGUGUCCCCGCUGCUGUGCAACGGUCUCCUGACUGGUGCUGCAGCACCGAGGGGCCUUUGUUCUGGGGGGACCCAGUGCAGAAUGUAAGUGGGCCCACCCGGUGGGACCCCCGUGGGGCAGGGAGCUGGGCCCGACAUGGCUCCGGCCUCUGCCUUUGCACCACGGGACAUCACAGGGUGGGCCUCGGCCCCUCCCACACCCAAAGCUGAGCCUGCAGGGAAGCCCCACAUGUCCAGCACCUUGUGCCUGGGGUGUUAGUGGCACCGCCUCCCCACCUCCAGGCUUUCCCACUUCCCACCCUGCCCCUCAGAGACUGAAAUUACGGGUACCUGAAGAUGGGAGCCUUUACCUUUUAUGCAAAAGGUUUAUUCCGGAAACUAGUGUACAUUUCUAUAAAUAGAUGCUGUGUAUAUGGUAUAUAUACAUAUAUAUAUAUAACAUAUAUGGAAGAGGAAAAGGCUGGUACAACGGAGGCCUGCGACCCUGGGGGCACAGGAGGCAGGCAUGGCCCUGGGCGGGGCGUGGGGGGGCGUGGAGGGAGGCCCCAGGGGGUCUCACCCAUGCAAGCAGAGGACCAGGGCCUUUUCUGGCACCGCAGUUUUGUUUUAAAACUGGACCUGUAUAUUUGUAAAGCUAUUUAUGGGCCCCUGGCACUCUUGUUCCCACACCCCAACACUUCCAGCAUUUAGCUGGCCACAUGGCGGAGAGUUUUAAUUUUUAACUUAUUGACAACCGAGAAGGUUUAUCCCGCCGAUAGAGGGACGGCCAAGAAUGUACGUCCAGCCUGCCCCGGAGCUGGAGGAUCCCCUCCAAGCCUAAAAGGUUGUUAAUAGUUGGAGGUGAUUCCAGUGAAGAUAUUUUAUUUCCUUUGUCCUUUUUCAGGAGAAUUAGAUUUCUAUAGGAUUUUUCUUUAGGAGAUUUAUUUUUUGGACUUCAAAGCAAGCUGGUAUUUUCAUACAAAUUCUUCUAAUUGCUGUGUGUCCCAGGCAGGGAGACGGUUUCCAGGGAGGGGCCGGCCCUGUGUGCAGGUUCCGAUGUUAUUAGAUGUUACAAGUUUAUAUAUAUCUAUAUAUAUAAUUUAUUGAGUUUUUACAAGAUGUAUUUGUUGUAGACUUAACACUUCUUACGCAAUGCUUCUAGAGUUUUAUAGCCUGGACUGCUACCUUUCAAAGCUUGGAGGGAAGCCGUGAAUUCAGUUGGUUCGUUCUGUACUGUUACUGGGCCCUGAGUCUGGGCAGCUGUCCCUUGCUUGCCUGCAGGGCCAUGGCUCAGGGUGGUCUCUUCUUGGGGCCCAGUGCAUGGUGGCCAGAGGUGUCACCCAAACCGGCAGGUGCGAUUUUGUUAACCCAGCGACGAACUUUCCGAAAAAUAAAGACACCUGGUUGCUAA | 0 (2) |
| EGF | AAACUGGAAUUAAAAGGAAAGUCAAGAAGAAUGAACUAUGUCGAUGCACAGUAUCUUUUCUUUCAAAAGUAGAGCAAAACUAUAGGUUUUGGUUCCACAAUCUCUACGACUAAUCACCUACUCAAUGCCUGGAGACAGAUACGUAGUUGUGCUUUUGUUUGCUCUUUUAAGCAGUCUCACUGCAGUCUUAUUUCCAAGUAAGAGUACUGGGAGAAUCACUAGGUAACUUAUUAGAAACCCAAAUUGGGACAACAGUGCUUUGUAAAUUGUGUUGUCUUCAGCAGUCAAUACAAAUAGAUUUUUGUUUUUGUUGUUCCUGCAGCCCCAGAAGAAAUUAGGGGUUAAAGCAGACAGUCACACUGGUUUGGUCAGUUACAAAGUAAUUUCUUUGAUCUGGACAGAACAUUUAUAUCAGUUUCAUGAAAUGAUUGGAAUAUUACAAUACCGUUAAGAUACAGUGUAGGCAUUUAACUCCUCAUUGGCGUGGUCCAUGCUGAUGAUUUUGCAAAAUGAGUUGUGAUGAAUCAAUGAAAAAUGUAAUUUAGAAACUGAUUUCUUCAGAAUUAGAUGGCUUAUUUUUUAAAAUAUUUGAAUGAAAACAUUUUAUUUUUAAAAUAUUACACAGGAGGCUUCGGAGUUUCUUAGUCAUUACUGUCCUUUUCCCCUACAGAAUUUUCCCUCUUGGUGUGAUUGCACAGAAUUUGUAUGUAUUUUCAGUUACAAGAUUGUAAGUAAAUUGCCUGAUUUGUUUUCAUUAUAGACAACGAUGAAUUUCUUCUAAUUAUUUAAAUAAAAUCACCAAAAACAUAAACAUUUUAUUGUAUGCCUGAUUAAGUAGUUAAUUAUAGUCUAAGGCAGUACUAGAGUUGAACCAAAAUGAUUUGUCAAGCUUGCUGAUGUUUCUGUUUUUCGUUUUUUUUUUUUUUCCGGAGAGAGGAUAGGAUCUCACUCUGUUAUCCAGGCUGGAGUGUGCAAUGGCACAAUCAUAGCUCAGUGCAGCCUCAAACUCCUGGGCUCAAGCAAUCCUCCUGCCUCAGCCUCCCGAGUAACUAGGACCACAGGCACAGGCCACCAUGCCUGGCUAAGGUUUUUAUUUUUAUUUUUUGUAGACAUGGGGAUCACACAAUGUUGCCCAGGCUGGUCUUGAACUCCUGGCCUCAAGCAAGGUCGUGCUGGUAAUUUUGCAAAAUGAAUUGUGAUUGACUUUCAGCCUCCCAACGUAUUAGAUUAUAGGCAUUAGCCAUGGUGCCCAGCCUUGUAACUUUUAAAAAAAUUUUUUAAUCUACAACUCUGUAGAUUAAAAUUUCACAUGGUGUUCUAAUUAAAUAUUUUUCUUGCAGCCAAGAUAUUGUUACUACAGAUAACACAACCUGAUAUGGUAACUUUAAAUUUUGGGGGCUUUGAAUCAUUCAGUUUAUGCAUUAACUAGUCCCUUUGUUUAUCUUUCAUUUCUCAACCCCUUGUACUUUGGUGAUACCAGACAUCAGAAUAAAAAGAAAUUGAAGUACCUGUUUUCAAAUGGAUACUUUAUAGGAAUUUUGGUAAAGAUUUGGUGAUGGGAGGAUGACUUGAGGUUUGUGGAUAUUAGUUAAUUAUUCAGUAUGAUACCUCACCCAGCUAAUUUAGAUUUUUCUAUAUUCGGUUUUGCUUUCAUUGACAAUAUCCUGGAGGAUCAGAAGACUUGUCUAUUUCUGCUGAGUCACUGGCCUCAGAAAAAUAAUAACCAUAAUUUCCCCCAAGGUUUUCUUUACCUAAGUGUGAAUAUUUUUUCUUCCUCCAAAAGCUCACUUUUGGGUUUAGAUUAAAUUUUUGUAUUUUAGCACCUUUUUCUUUUAGGGGUUCAAUGAUGACAAAAGAAAUGACAUGAGAACACGGCUACCCAUAACAUACCAUUAUCUUUGUACCAGAAAAAUCCUUGUUUCCUUCUUAAUGACUCUGGUACCUUAGAAACUGGGACCCUGCUAAGUCCUUGACUAGGCUAUCUACCAGCUCCUGGUCGGAUUAAAGAAAAAACACACUUUGUGUUUUUUAAUCACCAAGGCACCCUGCAGAGAUAUCUUCUUCUUGCAACUUCACAUCUUUAUCAGUAAUGUCCUCUUUCCUUUAAAAAUUCAAGUUUUAAGAACAGCAUUUUCAUGUAAAAACUUGAUUUGUGUUUUUUCCAGACUGAAUACUUUUCCUCCCUAACUCUCAUCGUCUCAUUGCGCGCAACGCCUGAUUGAGCUUCUGUUUGACUAAAUAUCACCUACUAUGUAAAAAAUGAGCAUAUUGGCCUCUUUUCUAGCAUCUAAUAAAGGCUUAAUACACUGUA | 0 (8) |
| MSX1 | AGGGUCCCAGGUCGCCCACCUGUGGGCCAGCCGAUUCCUCCAGCCCUGGUGCUGUACCCCCGACGUGCUCCCCUGCUCGGCACCGCCAGCCGCCUUCCCUUUAACCCUCACACUGCUCCAGUUUCACCUCUUUGCUCCCUGAGUUCACUCUCCGAAGUCUGAUCCCUGCCAAAAAGUGGCUGGAAGAGUCCCUUAGUACUCUUCUAGCAUUUAGAUCUACACUCUCGAGUUAAAGAUGGGGAAACUGAGGGCAGAGAGGUUAACAGAUUUAUCUAAGGUCCCCAGCAGAAUUGACAGUUGAACAGAGCUAGAGGCCAUGUCUCCUGCAUAGCUUUUCCCUGUCCUGACACCAGGCAAGAAAAGCGCAGAGAAAUCGGUGUCUGACGAUUUUGGAAAUGAGAACAAUCUCAAAAAAAAAAAAAAAAAAAAAAAAAAAAAAAAAAAAGAAAAGAGAAAAAAAAGACUAGCCAGCCAGGAAGAUGAAUCCUAGCUUCUUCCAUUGGAAAAUUUAAGACAAGUUCAACAACAAAACAUUUGCUCUGGGGGGCAGGGAAAACACAGAUGUGUUGCAAAGGUAGGUUGAAGGGACCUCUCUCUUACCAGUACCAGAAACACAAUUGUAAAAUUAAAAAAAAAAAAAAACUCUUUCUAUUUAACAGUACAUUUGUGUGGCUCUCAAACAUCCCUUUGGAAGGGAUUGUGUGUACUAUGUAAUAUACUGUAUAUUUGAAAUUUUAUUAUCAUUUAUAUUAUAGCUAUAUUUGUUAAAUAAAUUAAUUUUAAGCUACAAAAA | 0 (4) |
| ITGA2 | ACCAGCAGACCUACCUGCAGUGGGAACCGGCAGCAUCCCAGCCAGGGUUUGCUGUUUGCGUGAAUGGAUUUCUUUUUAAAUCCCAUAUUUUUUUUAUCAUGUCGUAGGUAAACUAACCUGGUAUUUUAAGAGAAAACUGCAGGUCAGUUUGGAAUGAAGAAAUUGUGGGGGGUGGGGGAGGUGCGGGGGGCAGGUAGGGAAAUAAUAGGGAAAAUACCUAUUUUAUAUGAUGGGGGAAAAAAAGUAAUCUUUAAACUGGCUGGCCCAGAGUUUACAUUCUAAUUUGCAUUGUGUCAGAAACAUGAAAUGCUUCCAAGCAUGACAACUUUUAAAGAAAAAUAUGAUACUCUCAGAUUUUAAGGGGGAAAACUGUUCUCUUUAAAAUAUUUGUCUUUAAACAGCAACUACAGAAGUGGAAGUGCUUGAUAUGUAAGUACUUCCACUUGUGUAUAUUUUAAUGAAUAUUGAUGUUAACAAGAGGGGAAAACAAAACACAGGUUUUUUCAAUUUAUGCUGCUCAUCCAAAGUUGCCACAGAUGAUACUUCCAAGUGAUAAUUUUAUUUAUAAACUAGGUAAAAUUUGUUGUUGGUUCCUUUUAGACCACGGCUGCCCCUUCCACACCCCAUCUUGCUCUAAUGAUCAAAACAUGCUUGAAUAACUGAGCUUAGAGUAUACCUCCUAUAUGUCCAUUUAAGUUAGGAGAGGGGGCGAUAUAGAGAAUAAGGCACAAAAUUUUGUUUAAAACUCAGAAUAUAACAUGUAAAAUCCCAUCUGCUAGAAGCCCAUCCUGUGCCAGAGGAAGGAAAAGGAGGAAAUUUCCUUUCUCUUUUAGGAGGCACAACAGUUCUCUUCUAGGAUUUGUUUGGCUGACUGGCAGUAACCUAGUGAAUUUCUGAAAGAUGAGUAAUUUCUUUGGCAACCUUCCUCCUCCCUUACUGAACCACUCUCCCACCUCCUGGUGGUACCAUUAUUAUAGAAGCCCUCUACAGCCUGACUUUCUCUCCAGCGGUCCAAAGUUAUCCCCUCCUUUACCCCUCAUCCAAAGUUCCCACUCCUUCAGGACAGCUGCUGUGCAUUAGAUAUUAGGGGGGAAAGUCAUCUGUUUAAUUUACACACUUGCAUGAAUUAC**UGUAUAUA**AACUCCUUAACUUCAGGGAGCUAUUUUCAUUUAGUGCUAAACAAGUAAGAAAAAUAAGCUCGAGUGAAUUUCUAAAUGUUGGAAUGUUAUGGGAUGUAAACAAUGUAAAGUAAGACAUCUCAGGAUUUCACCAGAAGUUACAGAUGAGGCACUGGAAGCCACCAAAUUAGCAGGUGCACCUUCUGUGGCUGUCUUGUUUCUGAAGUACUUAAACUUCCACAAGAGUGAAUUUGACCUAGGCAAGUUUGUUCAAAAGGUAGAUCCUGAGAUGAUUUGGUCAGAUUGGGAUAAGGCCCAGCAAUCUGCAUUUUAACAAGCACCCCAGUCACUAGGAUGCAGAUGGACCACACUUUGAGAAACACCACCCAUUUCUACUUUUUGCACCUUAUUUUCUCUGUUCCUGAGCCCCCACAUUCUCUAGGAGAAACUUAGAGGAAAAGGGCACAGACACUACAUAUCUAAAGCUUUGGACAAGUCCUUGACCUCUAUAAACUUCAGAGUCCUCAUUAUAAAAUGGGAAGACUGAGCUGGAGUUCAGCAGUGAUGCUUUUAGUUUUAAAAGUCUAUGAUCUGGACUUCCUAUAAUACAAAUACACAAUCCUCCAAGAAUUUGACUUGGAAAAAAAUGUCAAAGGAAAACAGGUUAUCUGCCCAUGUGCAUAUGGACAACCUUGACUACCCUGGCCUGGCCCGUGGUGGCAGUCCAGGGCUAUCUGUACUGUUUACAGAAUUACUUUGUAGUUGACAACACAAAACAAACAAAAAAGGCAUAAAAUGCCAGCGGUUUAUAGAAAAAACAGCAUGGUAUUCUCCAGUUAGGUAUGCCAGAGUCCAAUUCUUUUAACAGCUGUGAGAAUUUGCUGCUUCAUUCCAACAAAAUUUUAUUUAAAAAAAAAAAAAAAAGACUGGAGAAACUAGUCAUUAGCUUGAUAAAGAAUAUUUAACAGCUAGUGGUGCUGGUGUGUACCUGAAGCUCCAGCUACUUGAGAGACUGAGACAGGAAGAUCGCUUGAGCCCAGGAGUUCAAGUCCAGCCUAAGCAACAUAGCAAGACCCUGUCUCAAAAAAAUGACUAUUUAAAAAGACAAUGUGGCCAGGCACGGUGGCUCACACCUGUAAUCCCAACACUUUGGGAGGCUGAGGCCGGUGGAUCACGAGGUCAGGAGUUUGAGACUAGCCUGGCCAACAUGGUGAAACCCCAUCUCUAAUAAUAUAAAAAUUAGCUGGGCGUAGUAGCAGGUGCCUGUAAUCCCAGUUACUCGGGAAGCUGAGGCAGGAGAAUCACUUGAACCCGGGAGGCAGAGGUUUCAGUGAGCCGAGAUCGCGCCACUGCACUCCAGCCUGGGUGACAGGGCAAGACUCUGUCUCAAACAAACAAACAAAAAAAAAGUUAGUACUGUAUA**UGUAAAUA**CUAGCUUUUCAAUGUGCUAUACAAACAAUUAUAGCACAUCCUUCCUUUUACUCUGUCUCACCUCCUUUAGGUGAGUACUUCCUUAAAUAAGUGCUAAACAUACAUAUACGGAACUUGAAAGCUUUGGUUAGCCUUGCCUUAGGUAAUCAGCCUAGUUUACACUGUUUCCAGGGAGUAGUUGAAUUACUAUAAACCAUUAGCCACUUGUCUCUGCACCAUUUAUCACACCAGGACAGGGUCUCUCAACCUGGGCGCUACUGUCAUUUGGGGCCAGGUGAUUCUUCCUUGCAGGGGCUGUCCUGUACCUUGUAGGACAGCAGCCCUGUCCUAGAAGGUAUGUUUAGCAGCAUUCCUGGCCUCUAGCUACCCGAUGCCAGAGCAUGCUCCCCCCGCAGUCAUGACAAUCAAAAAAUGUCUCCAGACAUUGUCAAAUGCCUCCUGGGGGGCAGUAUUUCUCAAGCACUUUUAAGCAAAGGUAAGUAUUCAUACAAGAAAUUUAGGGGGAAAAAACAUUGUUUAAAUAAAAGCUAUGUGUUCCUAUUCAACAAUAUUUUUGCUUUAAAAGUAAGUAGAGGGCAUAAAAGAUGUCAUAUUCAAAUUUCCAUUUCAUAAAUGGUGUACAGACAAGGUCUAUAGAAUGUGGUAAAAACUUGACUGCAACACAAGGCUUAUAAAAUAGUAAGAUAGUAAAAUAGCUUAUGAAGAAACUACAGAGAUUUAAAAUUGUGCAUGACUCAUUUCAGCAGCAAAAUAAGAACUCCUAACUGAACAGAAAUUUUUCUACCUAGCAAUGUUAUUCUUGUAAAAUAGUUACCUAUUAAAACUGUGAAGAGUAAAACUAAAGCCAAUUUAUUAUAGUCACACAAGUGAUUAUACUAAAAAUUAUUAUAAAGGUUAUAAUUUUAUAAUGUAUUUACCUGUCCUGAUAUAUAGCUAUAACCCAAUAUAUGAAAAUCUCAAAAAUUAAGACAUCAUCAUACAGAAGGCAGGAUUCCUUAAACUGAGAUCCCUGAUCCAUCUUUAAUAUUUCAAUUUGCACACAUAAAACAAUGCCCUUUUGUGUACAUUCAGGCAUACCCAUUUUAAUCAAUUUGAAAGGUUAAUUUAAACCUCUAGAGGUGAAUGAGAAACAUGGGGGAAAAGUAUGAAAUAGGUGAAAAUCUUAACUAUUUCUUUGAACUCUAAAGACUGAAACUGUAGCCAUUA**UGUAAAUA**AAGUUUCAUAUGUACCUGUUUAUUUUGGCAGAUUAAGUCAAAAUAUGAA**UGUAUAUA**UUGCAUAACUAUGUUAGAAU**UGUAUAUA**UUUUAAAGAAAUUGUCUUGGAUAUUUUCCUUUAUACAUAAUAGAUAAGUCUUUUUUCAAAUGUGGUGUUUGAUGUUUUUGAUUAAAUGUGUUUUGCCUCUUUCCACAAAAACUGUAAAAAUAAAUGCAUGUUUGUACAAAAAGUUGCAGAAUUCAUUUGAUUUAUGAGAAACAAAAAUUAAAUUGUAGUCAACAGUUAGUAGUUUUUCUCAUAUCCAAGUAUAACAAACAGAAAAGUUUCAUUAUUGUAACCCACUUUUUUCAUACCACAUUAUUGAAUAUUGUUACAAUUGUUUUGAAAAUAAAGCCAUUUUCUUUGGGCUUUUAUAAGUUA | 5 (7) |
| FGFR2 | AUGACUGUGUCUGCCUGUCCCCAAACAGGACAGCACUGGGAACCUAGCUACACUGAGCAGGGAGACCAUGCCUCCCAGAGCUUGUUGUCUCCACU**UGUAUAUA**UGGAUCAGAGGAGUAAAUAAUUGGAAAAGUAAUCAGCAUAUGUGUAAAGAUUUAUACAGUUGAAAACUUGUAAUCUUCCCCAGGAGGAGAAGAAGGUUUCUGGAGCAGUGGACUGCCACAAGCCACCAUGUAACCCCUCUCACCUGCCGUGCGUACUGGCUGUGGACCAGUAGGACUCAAGGUGGACGUGCGUUCUGCCUUCCUUGUUAAUUUUGUAAUAAUUGGAGAAGAUUUAUGUCAGCACACACUUACAGAGCACAAAUGCAGUAUAUAGGUGCUGGAUGUA**UGUAAAUA**UAUUCAAAUUAUGUAUAAAUAUAUAUUAUAUAUUUACAAGGAGUUAUUUUUUGUAUUGAUUUUAAAUGGAUGUCCCAAUGCACCUAGAAAAUUGGUCUCUCUUUUUUUAAUAGCUAUUUGCUAAAUGCUGUUCUUACACAUAAUUUCUUAAUUUUCACCGAGCAGAGGUGGAAAAAUACUUUUGCUUUCAGGGAAAAUGGUAUAACGUUAAUUUAUUAAUAAAUUGGUAAUAUACAAAACAAUUAAUCAUUUAUAGUUUUUUUUGUAAUUUAAGUGGCAUUUCUAUGCAGGCAGCACAGCAGACUAGUUAAUCUAUUGCUUGGACUUAACUAGUUAUCAGAUCCUUUGAAAAGAGAAUAUUUACAAUAUAUGACUAAUUUGGGGAAAAUGAAGUUUUGAUUUAUUUGUGUUUAAAUGCUGCUGUCAGACGAUUGUUCUUAGACCUCCUAAAUGCCCCAUAUUAAAAGAACUCAUUCAUAGGAAGGUGUUUCAUUUUGGUGUGCAACCCUGUCAUUACGUCAACGCAACGUCUAACUGGACUUCCCAAGAUAAAUGGUACCAGCGUCCUCUUAAAAGAUGCCUUAAUCCAUUCCUUGAGGACAGACCUUAGUUGAAAUGAUAGCAGAAUGUGCUUCUCUCUGGCAGCUGGCCUUCUGCUUCUGAGUUGCACAUUAAUCAGAUUAGCCUGUAUUCUCUUCAGUGAAUUUUGAUAAUGGCUUCCAGACUCUUUGGCGUUGGAGACGCCUGUUAGGAUCUUCAAGUCCCAUCAUAGAAAAUUGAAACACAGAGUUGUUCUGCUGAUAGUUUUGGGGAUACGUCCAUCUUUUUAAGGGAUUGCUUUCAUCUAAUUCUGGCAGGACCUCACCAAAAGAUCCAGCCUCAUACCUACAUCAGACAAAAUAUCGCCGUUGUUCCUUCUGUACUAAAGUAUUGUGUUUUGCUUUGGAAACACCCACUCACUUUGCAAUAGCCGUGCAAGAUGAAUGCAGAUUACACUGAUCUUAUGUGUUACAAAAUUGGAGAAAGUAUUUAAUAAAACCUGUUAAUUUUUAUACUGACAAUAAAAAUGUUUCUACAGAUAUUAAUGUUAACAAGACAAAAUAAAUGUCACGCAACUUAUUUUUUUAA | 2 (4) |
| TGFBR2 | CUCUUCUGGGGCAGGCUGGGCCAUGUCCAAAGAGGCUGCCCCUCUCACCAAAGAACAGAGGCAGCAGGAAGCUGCCCCUGAACUGAUGCUUCCUGGAAAACCAAGGGGGUCACUCCCCUCCCUGUAAGCUGUGGGGAUAAGCAGAAACAACAGCAGCAGGGAGUGGGUGACAUAGAGCAUUCUAUGCCUUUGACAUUGUCAUAGGAUAAGCUGUGUUAGCACUUCCUCAGGAAAUGAGAUUGAUUUUUACAAUAGCCAAUAACAUUUGCACUUUAUUAAUGCC**UGUAUAUA**AAUAUGAAUAGCUAUGUUUUAUAUAUAUAUAUAUAUAUCUAUAUAUGUCUAUAGCUCUAUAUAUAUAGCCAUACCUUGAAAAGAGACAAGGAAAAACAUCAAAUAUUCCCAGGAAAUUGGUUUUAUUGGAGAACUCCAGAACCAAGCAGAGAAGGAAGGGACCCAUGACAGCAUUAGCAUUUGACAAUCACACAUGCAGUGGUUCUCUGACUGUAAAACAGUGAACUUUGCAUGAGGAAAGAGGCUCCAUGUCUCACAGCCAGCUAUGACCACAUUGCACUUGCUUUUGCAAAAUAAUCAUUCCCUGCCUAGCACUUCUCUUCUGGCCAUGGAACUAAGUACAGUGGCACUGUUUGAGGACCAGUGUUCCCGGGGUUCCUGUGUGCCCUUAUUUCUCCUGGACUUUUCAUUUAAGCUCCAAGCCCCAAAUCUGGGGGGCUAGUUUAGAAACUCUCCCUCAACCUAGUUUAGAAACUCUACCCCAUCUUUAAUACCUUGAAUGUUUUGAACCCCACUUUUUACCUUCAUGGGUUGCAGAAAAAUCAGAACAGAUGUCCCCAUCCAUGCGAUUGCCCCACCAUCUACUAAUGAAAAAUUGUUCUUUUUUUCAUCUUUCCCCUGCACUUAUGUUACUAUUCUCUGCUCCCAGCCUUCAUCCUUUUCUAAAAAGGAGCAAAUUCUCACUCUAGGCUUUAUCGUGUUUACUUUUUCAUUACACUUGACUUGAUUUUCUAGUUUUCUAUACAAACACCAAUGGGUUCCAUCUUUCUGGGCUCCUGAUUGCUCAAGCACAGUUUGGCCUGAUGAAGAGGAUUUCAACUACACAAUACUAUCAUUGUCAGGACUAUGACCUCAGGCACUCUAAACAUAUGUUUUGUUUGGUCAGCACAGCGUUUCAAAAAGUGAAGCCACUUUAUAAAUAUUUGGAGAUUUUGCAGGAAAAUCUGGAUCCCCAGGUAAGGAUAGCAGAUGGUUUUCAGUUAUCUCCAGUCCACGUUCACAAAAUGUGAAGGUGUGGAGACACUUACAAAGCUGCCUCACUUCUCACUGUAAACAUUAGCUCUUUCCACUGCCUACCUGGACCCCAGUCUAGGAAUUAAAUCUGCACCUAACCAAGGUCCCUUGUAAGAAAUGUCCAUUCAAGCAGUCAUUCUCUGGGUAUAUAAUAUGAUUUUGACUACCUUAUCUGGUGUUAAGAUUUGAAGUUGGCCUUUUAUUGGACUAAAGGGGAACUCCUUUAAGGGUCUCAGUUAGCCCAAGUUUCUUUUGCUUAUAUGUUAAUAGUUUUACCCUCUGCAUUGGAGAGAGGAGUGCUUUACUCCAAGAAGCUUUCCUCAUGGUUACCGUUCUCUCCAUCAUGCCAGCCUUCUCAACCUUUGCAGAAAUUACUAGAGAGGAUUUGAAUGUGGGACACAAAGGUCCCAUUUGCAGUUAGAAAAUUUGUGUCCACAAGGACAAGAACAAAGUAUGAGCUUUAAAACUCCAUAGGAAACUUGUUAAUCAACAAAGAAGUGUUAAUGCUGCAAGUAAUCUCUUUUUUAAAACUUUUUGAAGCUACUUAUUUUCAGCCAAAUAGGAAUAUUAGAGAGGGACUGGUAGUGAGAAUAUCAGCUCUGUUUGGAUGGUGGAAGGUCUCAUUUUAUUGAGAUUUUUAAGAUACAUGCAAAGGUUUGGAAAUAGAACCUCUAGGCACCCUCCUCAGUGUGGGUGGGCUGAGAGUUAAAGACAGUGUGGCUGCAGUAGCAUAGAGGCGCCUAGAAAUUCCACUUGCACCGUAGGGCAUGCUGAUACCAUCCCAAUAGCUGUUGCCCAUUGACCUCUAGUGGUGAGUUUCUAGAAUACUGGUCCAUUCAUGAGAUAUUCAAGAUUCAAGAGUAUUCUCACUUCUGGGUUAUCAGCAUAAACUGGAAUGUAGUGUCAGAGGAUACUGUGGCUUGUUUUGUUUAUGUUUUUUUUUCUUAUUCAAGAAAAAAGACCAAGGAAUAACAUUCUGUAGUUCCUAAAAAUACUGACUUUUUUCACUACUAUACAUAAAGGGAAAGUUUUAUUCUUUUAUGGAACACUUCAGCUGUACUCAUGUAUUAAAAUAGGAAUGUGAAUGCUAUAUACUCUUUUUAUAUCAAAAGUCUCAAGCACUUAUUUUUAUUCUAUGCAUUGUUUGUCUUUUACAUAAAUAAAAUGUUUAUUAGAUUGAAUAAAGCAAAAUACUCAGGUGAGCAUCCUGCCUCCUGUUCCCAUUCCUAGUAGCUAAA | 1 (1) |

**Table S2. List of primers used in the present study.**

| **Primers** | **Sequence or Cat. No. of validated primers** |
| --- | --- |
|  |  |
| *β-ACTIN* | 5′-GTCCTCTCCCAAGTCCACACA-3′ (sense, NM_001101.3)  5′-GGGCACGAAGGCTCATCATTC-3′ (antisense) |
| *PUM1* | Cat. No- P201905 V (RNA accession: NM_001020658.1)  <https://www.bioneer.co.kr/sirna-customorder.html> |
| *PUM2* | Cat. No- P130415 V (RNA accession: NM_001282752.2)  <https://www.bioneer.co.kr/sirna-customorder.html> |
| *DLX5* | Cat. No- P199945 V (RNA accession: NM_005221.5)  <https://www.bioneer.co.kr/sirna-customorder.html> |
| *FGFR3* | Cat. No- P106612 V (RNA accession: NM_000142.4)  <https://www.bioneer.co.kr/sirna-customorder.html> |
| *EGF* | Cat. No- P120876 V (RNA accession: NM_001178130.2)  <https://www.bioneer.co.kr/sirna-customorder.html> |
| *MSX1* | Cat. No- P174756 V (RNA accession: NM_002448.3)  <https://www.bioneer.co.kr/sirna-customorder.html> |
| *ITGA2* | Cat. No- P325457 V (RNA accession: NM_002203.3)  <https://www.bioneer.co.kr/sirna-customorder.html> |
| *FGFR2* | Cat. No- P100775 V (RNA accession: NM_000141.4)  <https://www.bioneer.co.kr/sirna-customorder.html> |
| *TGFBR2* | Cat. No- P260529 V (RNA accession: NM_001024847.2)  <https://www.bioneer.co.kr/sirna-customorder.html> |

**Table S3. siRNAs used in the current study.**

| **siRNAs** | **Dose**  **(nM)** | | **Sequence or Cat. No of validated siRNAs** |
| --- | --- | --- | --- |
| Negative control | | 100 | Sense siRNA targeted sequence 5-CCUACGCCACCAAUUUCGU-3′  antisense siRNA targeted sequence 5-ACGAAAUUGGUGGCGUAGG-3′ |
| *Human PUM1* | | 100 | siRNA ID- 9698-1 (Bioneer; https://www.bioneer.co.kr/sirna-customorder.html) |
| *Human PUM2* | | 100 | siRNA ID- 23369-1 (Bioneer; https://www.bioneer.co.kr/sirna-customorder.html) |
| *Rat Pum2* | | 100 | siRNA ID- 298874-1 (Bioneer; https://www.bioneer.co.kr/sirna-customorder.html) |
